# CD109-GP130 interaction drives glioblastoma stem cell plasticity and chemoresistance through STAT3 activity

**DOI:** 10.1172/jci.insight.141486

**Published:** 2021-05-10

**Authors:** Pauliina Filppu, Jayendrakishore Tanjore Ramanathan, Kirsi J. Granberg, Erika Gucciardo, Hannu Haapasalo, Kaisa Lehti, Matti Nykter, Vadim Le Joncour, Pirjo Laakkonen

**Affiliations:** 1 Translational Cancer Medicine Research Program, Faculty of Medicine, University of Helsinki, Helsinki, Finland.; 2 Faculty of Medicine and Health Technology, Tampere University, Tampere, Finland.; 3 Science Center, Tampere University Hospital, Tampere, Finland.; 4 Individualized Drug Therapy Program, Research Programs Unit, Faculty of Medicine, University of Helsinki, Helsinki, Finland.; 5 Department of Pathology, Fimlab Laboratories, Tampere University Hospital and University of Tampere, Tampere, Finland.; 6 Department of Microbiology, Tumor and Cell Biology, Karolinska Institutet, Stockholm, Sweden.; 7 Department of Biomedical Laboratory Science, Faculty of Natural Sciences, Norwegian University of Science and Technology, Trondheim, Norway.; 8 Laboratory Animal Centre, Helsinki Institute of Life Science (HiLIFE), University of Helsinki, Helsinki, Finland.

**Keywords:** Oncology, Stem cells, Brain cancer, Molecular biology, Molecular pathology

## Abstract

Glioma stem cells (GSCs) drive propagation and therapeutic resistance of glioblastomas, the most aggressive diffuse brain tumors. However, the molecular mechanisms that maintain the stemness and promote therapy resistance remain poorly understood. Here we report CD109/STAT3 axis as crucial for the maintenance of stemness and tumorigenicity of GSCs and as a mediator of chemoresistance. Mechanistically, CD109 physically interacts with glycoprotein 130 to promote activation of the IL-6/STAT3 pathway in GSCs. Genetic depletion of CD109 abolished the stemness and self-renewal of GSCs and impaired tumorigenicity. Loss of stemness was accompanied with a phenotypic shift of GSCs to more differentiated astrocytic-like cells. Importantly, genetic or pharmacologic targeting of CD109/STAT3 axis sensitized the GSCs to chemotherapy, suggesting that targeting CD109/STAT3 axis has potential to overcome therapy resistance in glioblastoma.

## Introduction

Glioblastoma, the most common malignant primary brain tumor, accounts for 14.5% of all tumors and almost half of the malignant tumors of the CNS (1). The standard of care includes maximal surgical resection followed by treatment with radiation and temozolomide (TMZ) chemotherapy. Despite these efforts, median survival is only approximately 15 months (2). Glioblastomas are highly heterogeneous tumors with at least 3 identified transcriptional subtypes within the isocitrate dehydrogenase (*IDH*) WT glioblastomas: mesenchymal (MES), classical (CL), and proneural (PN) (3), which can coexist within a single tumor (4).

Glioblastoma stem cells (GSCs) can self-renew, drive tumorigenesis, and promote tumor recurrence following chemotherapy (5–7). GSCs are highly plastic with a capacity for adaptation that fuels tumor heterogeneity and therapy resistance (8–10). For example, treatment of glioblastoma cells with TMZ induces a phenotypic shift of differentiated tumor cells to a stem-like state (11). Thus, GSCs are a key therapeutic target, but effective therapies have remained elusive. Therefore, the molecular underpinnings driving GSCs’ plasticity, tumorigenicity, and therapy resistance must be elucidated with potential implications for therapy.

CD109 is a glycosylphosphatidylinositol-anchored (GPI-anchored) glycoprotein of the α2-macroglobulin/complement family (12), classically known as a negative regulator of the TGF-β signaling in human keratinocytes (13, 14). In nonpathologic conditions, CD109 is suspected to play roles in skin organization (15) and osteogenesis (16) based on studies with CD109-deficient mouse models. To understand the role of CD109 in cancer, we used whole cell membrane proteomics and identified the cell surface CD109 as a metastasis-associated protein in breast cancers and melanomas (17). Moreover, CD109 has been shown to be overexpressed in many neoplasms, including brain tumors, and to promote lung cancer metastasis via the STAT3 activity (18–20). More specifically in gliomas, CD109 has been proposed as a marker of perivascular GSCs (20), and very recent reports indicate a clear association between CD109 and GSC stemness maintenance and disease recurrence (21, 22). However, the precise mechanisms of how CD109 regulates these phenomena and implications for GSC biology remained unknown.

Here, we describe a clinically relevant association of CD109 with the STAT3 activation in glioma samples and patient-derived GSCs. We demonstrate a physical interaction between CD109 and glycoprotein 130 (GP130) that is required for activation of the IL-6/STAT3 signaling pathway in GSCs. Sustained activation of the CD109/STAT3 axis is vital for the self-renewal and tumorigenicity of GSCs. Genetic or pharmacologic targeting of the CD109/STAT3 axis reprogrammed the GSCs to a chemosensitive state. Our results suggest that targeting the CD109/STAT3 axis in GSCs could provide means to overcome therapeutic resistance and improve treatment efficacy in patients with glioblastoma.

## Results

### CD109 associates with STAT3 phosphorylation and poor survival of malignant gliomas.

**Figure 1:**
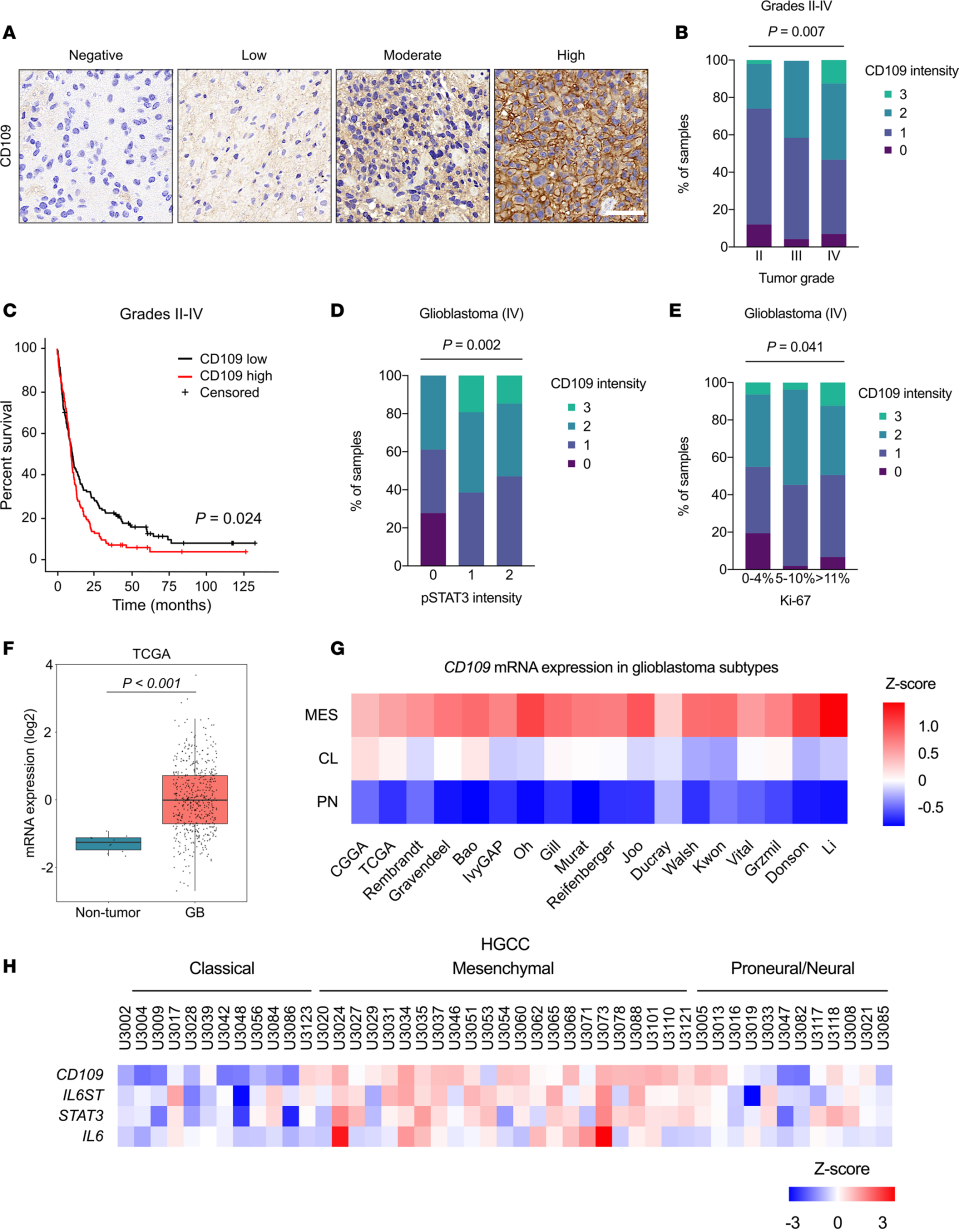
CD109 is associated with STAT3 phosphorylation and malignancy in glioblastoma. (**A**) Representative micrographs of negative, low, moderate, and high CD109 IHC staining of clinical glioma samples. Scale bar: 50 μm. (**B**) Association of CD109 expression with tumor grade (*n* = 346). *P* < 0.007, χ^2^ test. See also Supplemental Table 1. (**C**) Kaplan-Meier survival analysis based on CD109 expression. The median value was used as cutoff; CD109^hi^ (*n* = 118), red line, and CD109^lo^ (*n* = 122), black line. *P* = 0.024, log-rank test. (**D** and **E**) Association of CD109 expression with p-STAT3 (*n* = 78; *P* = 0.002) (**D**) and Ki-67 (*n* = 173; *P* = 0.041) (**E**) in glioblastomas, χ^2^ test. See also Supplemental Table 3. (**F**) *CD109* mRNA expression between glioblastoma (*n* = 489) and nontumor specimens (*n* = 10) in the TCGA glioblastoma (TCGA GBM) data set. *P* < 0.001, Tukey’s post hoc test. (**G**) Heatmap shows *CD109* mRNA expression between different glioblastoma subtypes across several glioblastoma data sets: mesenchymal (MES), classical (CL), and proneural (PN). (**H**) Heatmap shows mRNA expression levels of *CD109*, *IL6ST*, *STAT3*, and *IL-6* between glioblastoma cell lines across different subtypes in the HGCC data set.

To study the possible clinical relevance of CD109, we performed immunohistochemical (IHC) analysis of a large cohort of clinical astrocytoma tumor microarray specimens (grades II–IV; *n* = 346). Evaluation of the immunostaining revealed that CD109 is located at the plasma membrane of tumor cells in nearly all specimens (92.5%) (Figure 1A). CD109 was significantly overexpressed in glioblastomas (grade IV) compared with lower grade astrocytomas (grades II–III) (*P* = 0.007) (Figure 1B and Supplemental Table 1; supplemental material available online with this article; https://doi.org/10.1172/jci.insight.141486DS1). Kaplan-Meier survival analysis demonstrated that high CD109 expression associated with poorer survival (*P* = 0.024) of patients with increasingly malignant, diffusively infiltrating astrocytomas (grades II–IV) (Figure 1C). *IDH1* mutation status was available for 205 specimens, of which 86.8% were *IDH1* WT and 13.2% *IDH1* mutant tumors. We found no association between CD109 and the *IDH1* mutation, p53, or EGFR (Supplemental Table 2). However, further analyses of the patient samples revealed that CD109 expression significantly associated with increasingly phosphorylated STAT3 (p-STAT3, Tyr705) (*P* = 0.004) and higher number of Ki-67–positive, proliferating tumor cells (*P* = 0.027) (Supplemental Table 2). We then stratified the patient data by classifying gliomas into either lower grade astrocytomas (grades II–III) or glioblastomas (grade IV). The analysis of these groups revealed significant association between CD109 expression and p-STAT3 (*P* = 0.002) and Ki-67 (*P* = 0.041) only in glioblastomas (Figure 1, D and E, and Supplemental Table 3). Of the other possible associations, a significant association was detected between CD109 expression and tumor grade (*P* = 0.0002) (Table 1 and Supplemental Table 1). In brief, high CD109 expression is a biomarker of malignant glioblastomas, correlating with an increased activity of the STAT3 pathway and highly proliferating glioblastoma cells (Table 1).

To link our results with global studies, we interrogated publicly available data sets accessed via the GlioVis data portal (23). Analysis of The Cancer Genome Atlas (TCGA) glioblastoma data set demonstrated increased *CD109* mRNA expression in glioblastomas compared with the nontumor tissue, supporting the relationship between CD109 and tumorigenicity (Figure 1F). In accordance with our findings, high *CD109* mRNA expression associated with poorer survival in the data sets of lower grade gliomas (TCGA LGG) and when glioblastoma and lower grade gliomas were combined, but not in glioblastomas alone (TCGA GBM) (Supplemental Figure 1, A–C).

It has been recently suggested that CD109 expression associates with the MES glioblastoma subtype in TCGA data set (24). To profile CD109 expression within glioblastoma subtypes, we analyzed 17 individual glioblastoma data sets where the subtype-specific information was available, consisting of 2100 patient samples in total. The highest *CD109* mRNA levels were consistently detected in the MES subtype tumors followed by the CL and PN subtypes, respectively (Figure 1G).

MES glioblastoma subtype has been linked to increased infiltration of tumor-associated microglia and macrophages (3). Since the mRNA data derive from tumor tissue specimens, it may contain tumor-associated stromal cells and cannot discriminate gene expression information between tumor cells and the tumor-associated stromal cells. Therefore, we performed an analysis of the mRNA expression of *CD109* and the key mediators of the STAT3 signaling pathway (*IL6ST*/GP130, *STAT3*, and *IL6*) in the Human Glioblastoma Cell Culture (HGCC) data set (25). Consistently, we found the highest expression of analyzed genes in the MES glioblastoma cell lines (Figure 1H). Furthermore, analysis of the gene expression data from the human brain tumor immune microenvironment (26) demonstrated the highest *CD109* expression in the monocyte-derived macrophages, yet the expression did not increase in glioblastomas compared with the nontumor reference samples (Supplemental Figure 1D). These data demonstrate that CD109 expression is specific to tumor cells and associates particularly with the highly aggressive MES subtype.

### CD109/STAT3 axis fuels glioblastoma cell stemness.

**Figure 2:**
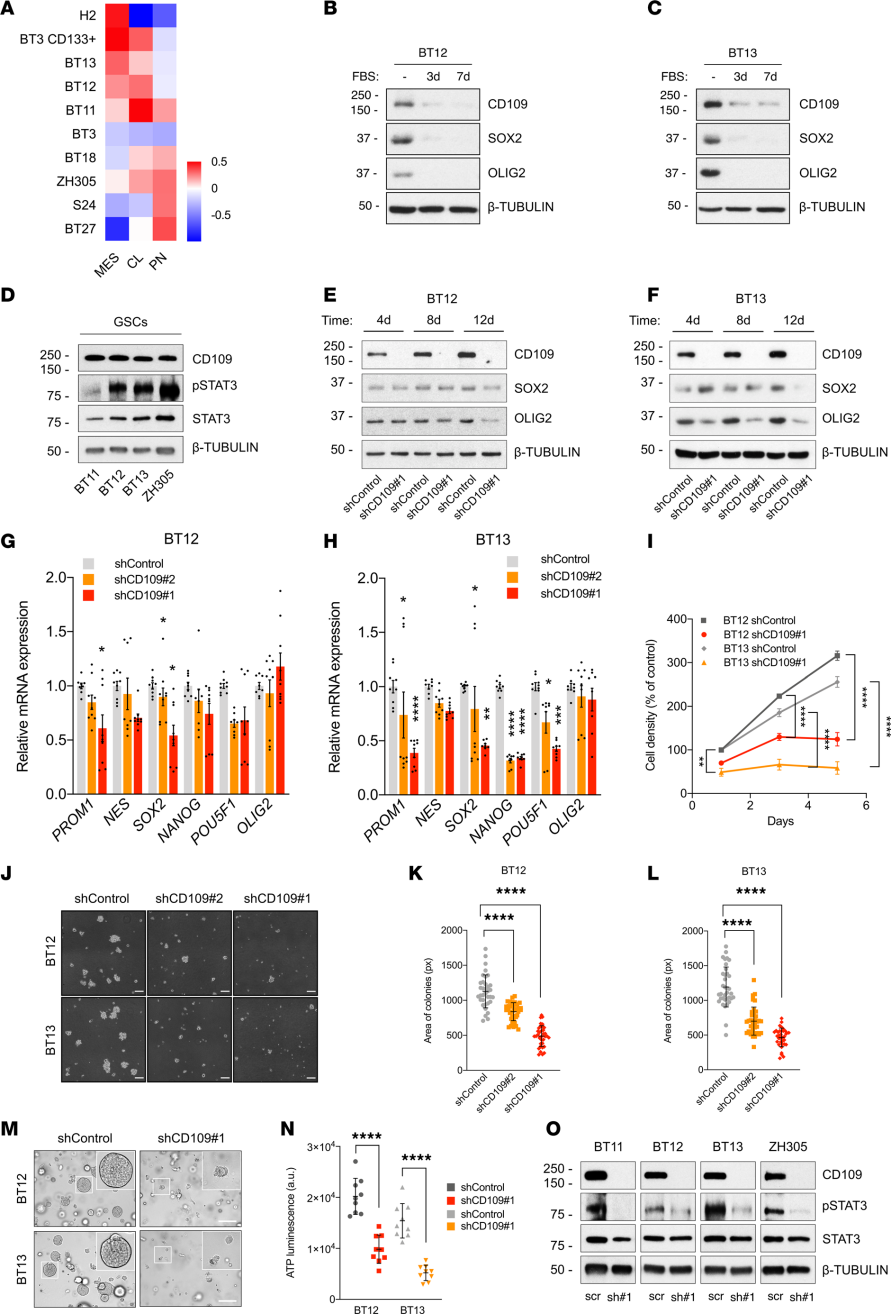
CD109/STAT3 axis maintains glioblastoma cell stemness. (**A**) Heatmap shows the classification of patient-derived GSCs and the H2 cell line based on their dominant transcriptional subtype: mesenchymal-like (MES), classical-like (CL), and proneural-like (PN). (**B** and **C**) Western blot analyses of CD109, SOX2, and OLIG2 expression in GSCs and differentiated glioma cells cultured in FBS-containing medium at the indicated time points. (**D**) Western blot analysis of CD109 expression and p-STAT3 levels in GSCs of different glioblastoma subtypes. (**E** and **F**) Western blot analyses of SOX2 and OLIG2 expression in CD109-silenced GSCs and nontargeted controls at the indicated time points. (**G** and **H**) Real-time quantitative PCR (qRT-PCR) analysis of GSC marker mRNA levels in CD109-silenced and nontargeted control GSCs at day 11. (**I**) Cell proliferation of CD109-silenced and nontargeted control GSCs at indicated time points. Data are presented as mean ± SEM. *P < 0.05; **P < 0.01; ****P < 0.0001, 2-way ANOVA with Tukey’s multiple comparisons test. (**J**) Representative micrographs of colony formation in methylcellulose after CD109 silencing. Scale bar: 200 μm. (**K** and **L**) Quantification of colonies in methylcellulose. Data are presented as mean ± SD. ****P < 0.0001, 1-way ANOVA with Dunnett’s multiple comparisons test. (**M**) Representative micrographs of GSC growth in 3D fibrin matrix after CD109 silencing. Scale bar: 200 μm. Inset: original magnification, ×10. See also Supplemental Figure 2D. (**N**) Cell viability in 3D fibrin matrix at day 15. Data are presented as mean ± SD. ****P < 0.0001, unpaired 2-tailed *t* test. See also Supplemental Figure 2E. (**O**) Western blot analysis of p-STAT3 levels after CD109 silencing. Data are from *n* = 2 (**B** and **C**) and *n* = 3 (**D**–**O**) independent experiments. Representative Western blots are shown where β-tubulin served as a loading control.

We generated patient-derived glioblastoma cell lines and cultured them as gliospheres in a serum-free medium that enriches GSCs and demonstrated that the GSCs were able to self-renew, initiate tumors upon orthotopic xenotransplantation, and recapitulate the cellular heterogeneity present in the original tumor (27), therefore fulfilling the functional criteria of GSCs (6, 28). To identify the gene expression profiles of the GSCs, we performed RNA sequencing (RNA-Seq) and gene expression analysis based on the recently published gene signatures (10). In this study, single-glioblastoma cells were found to exist in 4 main cellular states: mesenchymal-like (MES-like), astrocytic-like (AC-like), oligodendrocyte-progenitor-like (OPC-like), and neural-progenitor-like (NPC-like) (10). Enriched MES- and AC-like states in tumors correspond to the MES and CL glioblastoma subtypes, respectively, whereas the NPC- and OPC-like states together define the PN subtype (10). Consistent with the previous reports (4, 10), our cell lines displayed a combination of 2 or more cellular states. Therefore, we classified the cell lines based on their dominant transcriptional subtypes as follows: H2, BT3 CD133^+^, BT13, and BT12 as MES-like and BT18, ZH305, S24, and BT27 as PN-like. The MES-like signature tended to co-occur with the AC-like signature. Finally, BT11 and BT3 cells were classified as hybrids with no marked subtype identity (Figure 2A).

CD109 was highly expressed in the GSCs together with the GSC-associated transcription factors, such as SOX2 and oligodendrocyte transcription factor 2 (OLIG2) (29). Serum-induced cell differentiation caused a rapid and global downregulation of the GSC markers and CD109 (Figure 2, B and C), supporting the association between CD109 and glioblastoma cell stemness. In addition, STAT3 has been implicated in the stemness maintenance of GSCs (30) and was found to be constitutively phosphorylated in our patient-derived GSCs (Figure 2D).

To evaluate the dependency of GSCs of CD109, we silenced CD109 expression using lentiviral delivery of shRNA. Silencing efficiencies were confirmed at both mRNA and protein levels. The shCD109#1 showed highly efficient silencing of CD109 expression, whereas silencing efficiency with the shCD109#2 was partial (Supplemental Figure 2, A–C). Our results demonstrate decreased SOX2 and OLIG2 protein expression in response to CD109 silencing (Figure 2, E and F). Consistent with the decreased protein levels, several GSC-associated marker genes were also downregulated in CD109-silenced GSCs compared with the nontargeted controls (Figure 2, G and H), although the effect was more profound in the BT13 cells. In agreement with the loss of stemness, CD109 silencing significantly decreased cell proliferation (Figure 2I) and self-renewal capacity in a semisolid methylcellulose matrix in vitro when compared with the nontargeted control cells (Figure 2, J–L). Furthermore, CD109-silenced GSCs released significantly less ATP as a sign of reduced cell viability, and their growth was inhibited as demonstrated by the reduced size and highly disorganized structure of the gliospheres in a 3D fibrin matrix that more closely recapitulates mechanical properties of the brain tissue (Figure 2, M and N; and Supplemental Figure 2, D and E).

CD109-silenced GSCs showed markedly reduced p-STAT3 levels compared with the nontargeted controls independently of the glioblastoma subtype (Figure 2O), thus verifying the observed association between CD109 and p-STAT3 in the patient samples. Together these results demonstrate a vital role for CD109/STAT3 axis in the maintenance of glioblastoma cell stemness.

### CD109-GP130 physical interaction regulates IL-6/STAT3 pathway activation.

**Figure 3:**
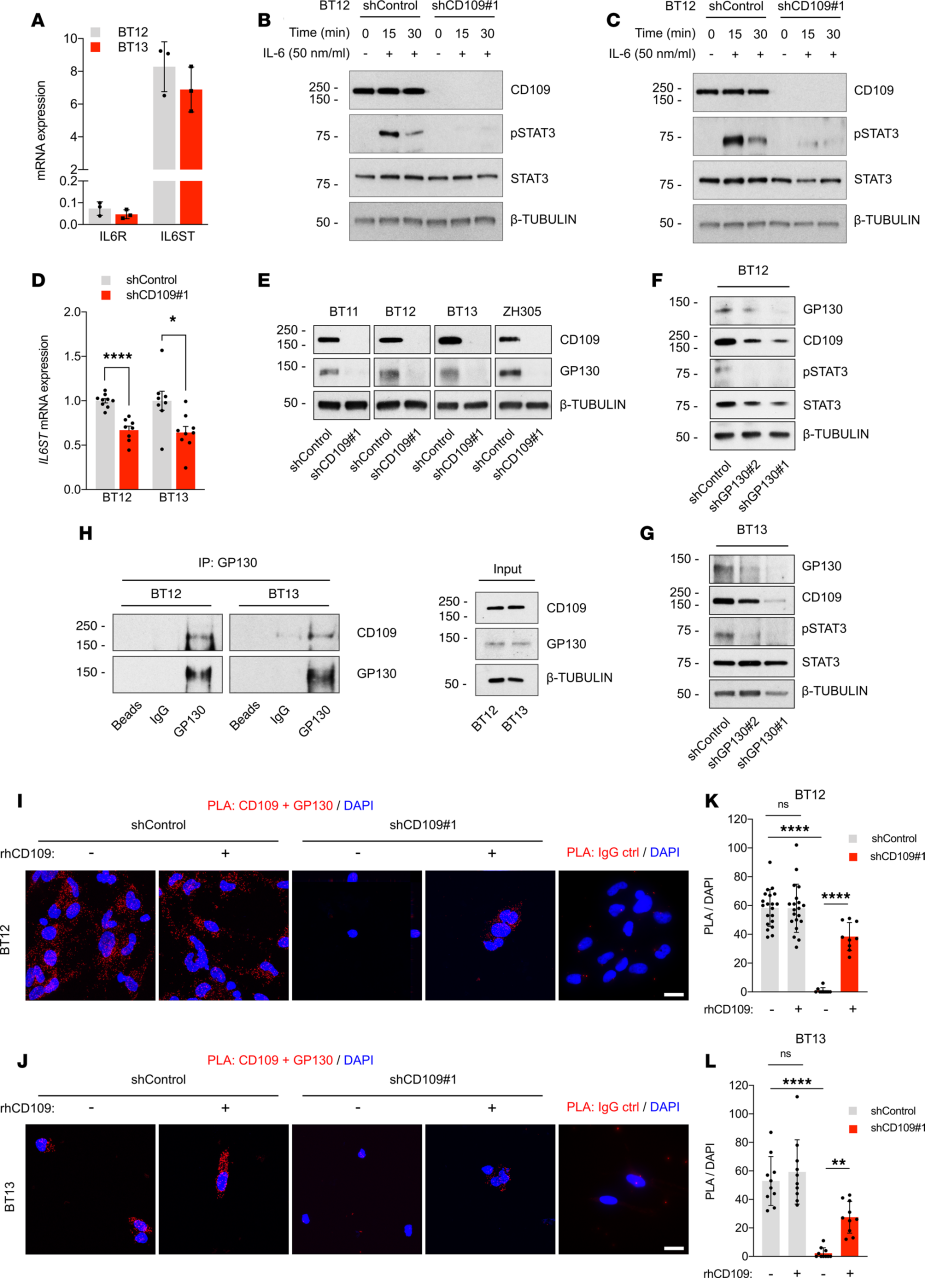
CD109-GP130 interaction promotes activation of the STAT3 pathway. (**A**) qRT-PCR analysis of *IL6R* and *IL6ST* mRNA levels in GSCs. Data are presented as mean ± SD. (**B** and **C**) Western blot analyses of p-STAT3 levels in CD109-silenced and nontargeted control GSCs after stimulation with IL-6 cytokine (50 ng/mL) for 15 and 30 minutes. (**D**) qRT-PCR analysis of *IL6ST* mRNA levels in CD109-silenced and nontargeted control GSCs. Data are presented as mean ± SEM. *P < 0.05; ****P < 0.0001, unpaired 2-tailed *t* test. (**E**) Western blot analysis of GP130 expression after CD109 silencing in GSCs of different glioblastoma subtypes. (**F** and **G**) Western blot analyses of CD109 expression and p-STAT3 levels in GP130-silenced and nontargeted control GSCs. (**H**) Co-IP analysis of CD109. GSC whole-cell extracts were subjected to immunoprecipitation with an anti-GP130 antibody or appropriate IgG control followed by Western blotting with an anti-CD109 antibody. Inputs are indicated. (**I** and **J**) Representative micrographs of PLA in GSCs using anti-CD109 and anti-GP130 antibodies. Appropriate IgG controls served as negative controls. Red indicates specific interaction signal. Nuclei were counterstained with DAPI (blue). Scale bar: 20 μm. (**K** and **L**) Quantification of PLA signal per cell (DAPI). Data are presented as mean ± SD. **P < 0.01; ****P < 0.0001, 1-way ANOVA with Tukey’s multiple comparisons test. Data are from *n* = 3 (**A**–**G**) and *n* = 2 (**H**–**L**) independent experiments. Representative Western blots are shown, where β-tubulin served as a loading control.

IL-6/GP130 signaling is the central STAT3 activating pathway. IL-6 binds to the IL-6 receptor (IL-6R), which then dimerizes with the common coreceptor and signal transducer GP130 to initiate intracellular signaling via JAK/STAT3 (31). GP130 is expressed on all cells in the body whereas IL-6R expression is limited to only a few cell types, including hepatocytes and leukocytes (31). GSCs have been previously shown to express IL-6R and to respond to IL-6 stimulation by STAT3 activation (32). We found high expression of *IL6ST* mRNA (GP130) and low levels of *IL6R* mRNA in our GSCs (Figure 3A). Moreover, CD109 silencing impaired STAT3 activation while the nontargeted control cells rapidly responded to IL-6 stimulation by activating the IL-6/STAT3 pathway. This was not observed in the CD109-silenced GSCs (Figure 3, B and C).

Next, we analyzed GP130 expression in response to CD109 silencing and detected a significant reduction in GP130 transcript and protein levels (Figure 3, D and E). To verify the importance of GP130 in STAT3 activation in the GSCs, we silenced GP130 expression using lentiviral shRNAs (Supplemental Figure 3A). Silencing of GP130 substantially reduced p-STAT3 levels and was accompanied with decreased CD109 expression (Figure 3, F and G).

To gain mechanistic insight into CD109/STAT3 signaling in GSCs, we hypothesized that CD109 would physically interact with GP130. To address this hypothesis, we first performed a coimmunoprecipitation (co-IP) assay on total cell extracts. The endogenous CD109 was found to coprecipitate with GP130 in GSCs (Figure 3H). To verify the physical interaction, we used the proximity ligation assay (PLA) using antibodies against CD109 and GP130. A strong PLA signal was detected in the nontargeted control GSCs but was absent in the CD109-silenced GSCs (Figure 3, I–L).

CD109 consists of a large 180 kDa and a small GPI-linked, 25 kDa subunit. The newly synthesized CD109 core protein is linked to GPI anchor and glycosylated in the ER, followed by furinase cleavage into 180 kDa and 25 kDa in the trans-Golgi network. Finally, the protein complex is transported and expressed on the plasma membrane. The 180 kDa subunit can be shed and secreted whereas the 25 kDa remains linked to the plasma membrane (33). To investigate whether the secreted CD109 and/or the membrane-bound subunit could also physically interact with GP130, we repeated the PLA assay by treating the cells with recombinant soluble CD109 or by using an antibody specific to the 25 kDa subunit (11H3), respectively. No PLA signal was detected when the antibody against the 25 kDa subunit (11H3) was used, suggesting that only the 180 kDa subunit of CD109 interacts with the GP130 (Supplemental Figure 3B). However, PLA signal was detected after treatment of CD109-silenced GSCs with the recombinant soluble human CD109 (Figure 3, I–L), whereas treatment with IL-6 did not affect the CD109-GP130 interaction (Supplemental Figure 3B). Our results show that the 180 kDa subunit of CD109 interacts with GP130 to regulate the activation of the IL-6/STAT3 pathway.

### CD109 silencing induces a compensatory phenotypic shift of GSCs to AC-like state.

**Figure 4:**
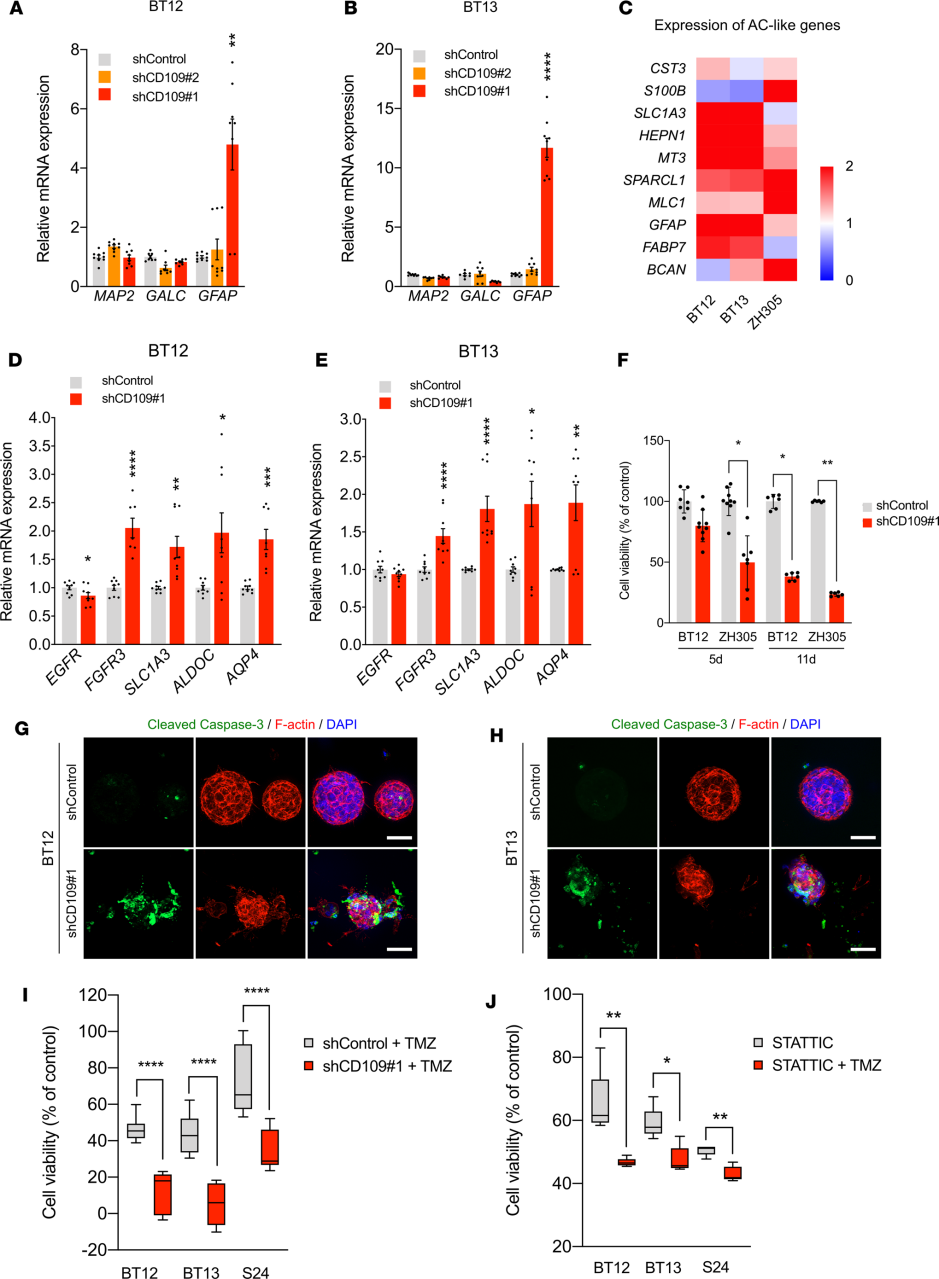
Loss of CD109 induces a compensatory phenotypic shift to an AC-like state and sensitizes the cells to chemotherapy. (**A** and **B**) qRT-PCR analysis of *MAP2*, *GALC*, and *GFAP* mRNA levels in CD109-silenced and nontargeted GSCs at day 11. Data are presented as mean ± SEM. **P < 0.01; ****P < 0.0001, 2-way ANOVA with Dunnett’s multiple comparisons test. (**C**) Heatmap shows increased expression of AC-like genes after CD109 silencing at day 11. Data are presented as fold change relative to control. (**D** and **E**) qRT-PCR analysis of CL subtype genes in CD109-silenced and nontargeted GSCs at d11. Data are presented as mean ± SEM. *P < 0.05; **P < 0.01; ***P < 0.001; ****P < 0.0001, unpaired 2-tailed *t* test. (**F**) Cell viability of CD109-silenced and nontargeted GSCs at the indicated time points. Data are presented as mean ± SD. *P < 0.05; **P < 0.01, 1-way ANOVA with Kruskal-Wallis post hoc test. (**G** and **H**) Immunofluorescence staining of cleaved caspase-3 (green) and F-actin (red) in CD109-silenced and nontargeting control GSCs in 3D fibrin matrix at day 15. Nuclei were counterstained with DAPI (blue). Scale bar: 100 μm. (**I**) Box-and-whisker plot shows cell viability of CD109-silenced and nontargeting control GSCs after treatment with 250 μM of TMZ for 4 days. Data were normalized to the corresponding vehicle control. ****P < 0.0001, nonparametric Mann-Whitney *U* test (BT12) and unpaired 2-tailed *t* test (BT13 and S24). (**J**) Box-and-whisker plot shows cell viability after treatment with Stattic or combination of Stattic and TMZ. Data were normalized to the corresponding vehicle control. *P < 0.05; **P < 0.01, nonparametric Mann-Whitney *U* test. Box plots represent the first quartile, median, and third quartile, with whiskers indicating minimum and maximum values. Data are from *n* = 3 (**A**, **B**, and **D**–**J**) independent experiments.

To investigate further the functional consequences of CD109 silencing, we first studied whether the loss of stemness was accompanied with increased GSC differentiation. qRT-PCR analysis of cell differentiation markers in CD109-silenced and nontargeted control GSCs revealed a significant increase in the mature astrocyte marker glial fibrillary acidic protein (*GFAP*) mRNA expression, whereas the levels of markers for mature neurons (microtubule associated protein 2, *MAP2*) or oligodendrocytes (galactosylceramidase, *GALC*) did not change (Figure 4, A and B). However, *GFAP* expression associates with the CL subtype of glioblastoma, and although GFAP remains the most used astrocyte marker, it is also regarded as a marker for reactive astrocytes and expressed by the neural progenitor cells (10, 34). To get a more comprehensive view of alterations in global gene expression profiles and their relationship with different glioblastoma subtypes, we profiled the control and CD109-silenced GSCs using RNA-Seq. Genes associated with the AC-like cellular state corresponding to the CL subtype were consistently upregulated in response to CD109 silencing (Figure 4C). We validated the expression of selected classical subtype genes (10, 35, 36) by qRT-PCR analysis. The mRNA levels of *FGFR3*, *SLC1A3*, *ALDOC*, and *AQP4* were significantly increased while *EGFR* mRNA level expression was slightly decreased in the CD109-silenced GSCs (Figure 4, D and E). Thus, CD109 silencing drives a phenotypic shift of the GSCs toward differentiated AC-like cells that corresponds to the CL subtype.

As STAT3 is a central driver of cell survival and inhibition of apoptosis in cancer (37), we determined the impact of CD109 silencing on cell viability. Measurement of the ATP levels released by metabolically active live cells revealed that the PN-like GSCs (ZH305) were significantly more sensitive to CD109 silencing than the MES-like GSCs (BT12) (Figure 4F). In accordance with this, the ZH305 cells showed a significant increase in cell death compared with the BT12 cells in vitro (Supplemental Figure 4, A–F). However, the detected significant growth inhibition of CD109-silenced MES-like GSCs in the 3D fibrin matrix also rendered the MES-like GSCs susceptible to apoptotic cell death (Figure 4, G and H).

### CD109 confers resistance to chemotherapy.

GSCs have been previously shown to be highly resistant to radiation and chemotherapy (8, 9). Due to their high plasticity, GSCs can dynamically adapt into the most fitting phenotypic state in response to therapy, therefore sustaining tumor progression (10, 38). Our results demonstrate that CD109 silencing significantly reduced GSC stemness and increased phenotypic shift to the AC-like state corresponding to the CL subtype. The CL subtype is generally associated with better response to treatment (38), therefore raising a question, whether CD109 targeting would sensitize the cells to TMZ chemotherapy. To address this question, we performed TMZ sensitivity tests on both MES-like (BT12 and BT13) and PN-like (S24) GSCs. Treatment of the control and CD109-silenced GSCs with 250 μM of TMZ for 4 days significantly reduced viability of the CD109-silenced MES-like GSCs compared with nontargeted control cells (Figure 4I). Even though the S24 cells showed high resistance to TMZ treatment, CD109 silencing significantly increased their sensitivity (Figure 4I).

Since our results demonstrate that CD109/STAT3 axis maintains GSC stemness and CD109-targeting agents are currently unavailable, we assessed blockage of the downstream signaling pathway by using a STAT3 inhibitor. We chose Stattic, which is a direct STAT3 inhibitor and has been previously shown to induce partial cell apoptosis in GSCs (32). Similar to CD109 silencing, treatment with Stattic sensitized the MES-like GSCs to TMZ. The sensitization effect was even more profound in the PN-like GSCs (Figure 4J). This proof of concept indicates that targeting CD109/STAT3 axis sensitizes the highly resistant GSCs to chemotherapy, supporting an important role for CD109 in conferring chemoresistance of glioblastoma.

### CD109 is vital for glioblastoma growth in vivo.

To investigate the role of CD109 in tumor initiation and progression in vivo, we analyzed the development of intracranial xenografts derived from control and CD109-silenced GSCs using 4 different sets of GSCs initially enriched with either the MES- or PN-like signature. At the experimental endpoint, after 25–35 days of tumor formation, we performed a comprehensive analysis of tumor progression, including measurement of the tumor volume and unbiased quantification of the invasive cells using the brain “snapshots” technique (Supplemental Figure 5, A and B). We also studied the role played by the tumor stroma, especially the microglial cells, by costaining of CD109 and ionized calcium-binding adapter molecule 1 (IBA-1) antibodies. Our observations reveal that the microglial component was a minority in the tumor compartment and CD109 was exclusively expressed by the tumor cells and not by the host microglia (Supplemental Figure 5, C–E).

**Figure 5:**
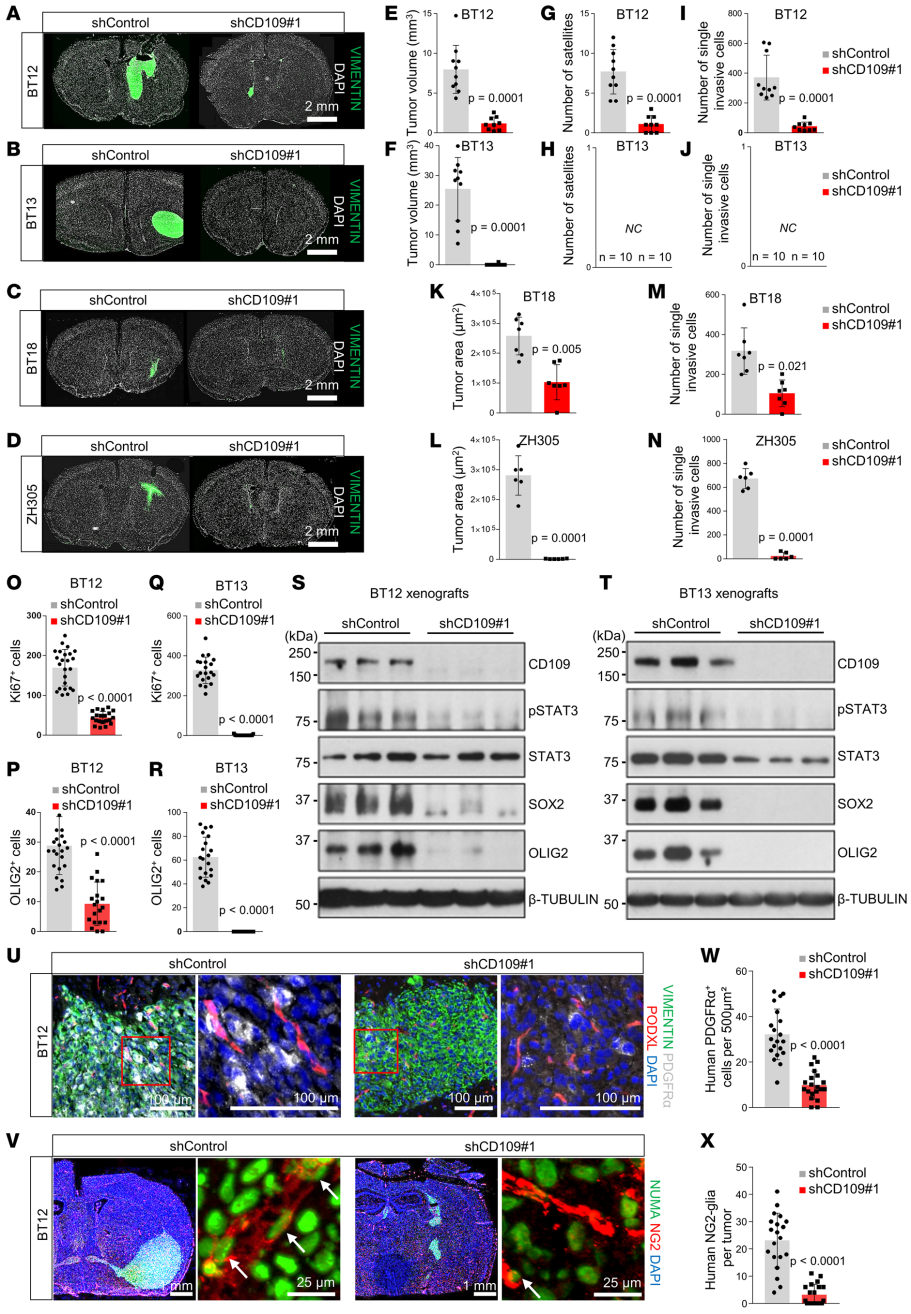
CD109 silencing profoundly hampers glioblastoma growth in vivo. Representative micrographs of coronal sections of tumor-bearing animal brains after implantation of nontargeted control or CD109-silenced BT12 (**A**), BT13 (**B**), BT18 (**C**), or ZH305 (**D**) GSCs. Frozen sections were immunostained with anti-human vimentin (green). Nuclei were counterstained with DAPI (white). Scale bar: 2 mm. Quantification of the BT12 (**E**) and BT13 (**F**) tumor volume, number of BT12 (**G**) and BT13 (**H**) satellite tumors, and number of single invasive BT12 (**I**) and BT13 (**J**) cells of control and CD109-silenced BT12 (*n* = 10) and BT13 (*n* = 10) xenografts. Quantification of the BT18 (**K**) and ZH305 (**L**) tumor area and number of single invasive BT18 (**M**) and ZH305 (**N**) cells of control and CD109-silenced BT18 (*n* = 7) and ZH305 (*n* = 6) xenografts. Quantification of the number of Ki-67^+^ BT12 (**O**) and BT13 (**P**) tumor cells and number or OLIG2^+^ BT12 (**Q**) and BT13 (**R**) tumor cells of control and CD109-silenced BT12 (*n* = 10) and BT13 (*n* = 10) xenografts. Western blot analysis of expression of indicated proteins from the whole brain section extracts of BT12 (**S**) and BT13 (**T**) control and CD109-silenced BT12 (*n* = 3) and BT13 (*n* = 3) xenografts. (**U**) Representative micrographs of BT12 xenografts immunostained with anti–PDGFR-α (white), anti-human vimentin (green), and podocalyxin (red). Nuclei were counterstained with DAPI (blue). Scale bar: 100 μm. (**V**) Representative micrographs of BT12 xenografts immunostained with anti-NG2 (red, arrows) and anti-human nuclear marker (NUMA, green). Nuclei were counterstained with DAPI (blue). Scale bars: 1 mm or 25 μm as indicated. (**W**) Quantification of the number of PDGFR-α^+^ cells in control and CD109-silenced BT12 (*n* = 10) xenografts. (**X**) Quantification of the number of NG2^+^ cells in control and CD109-silenced BT12 (*n* = 10) xenografts. *P* values were calculated using unpaired 2-tailed *t* test.

CD109 silencing significantly impaired tumor growth in all studied models (Figure 5, A–N). MES-like BT12 displays a multimodal growth with the formation of a large primary tumor and smaller satellite tumors accompanied with extensive collective migration along the blood capillaries and single-cell invasion into the brain parenchyma (27). CD109 silencing significantly reduced tumor size (Figure 5, A and E) and decreased the number of satellite tumors (Figure 5G) and invading single cells (Figure 5I) compared with the control tumors. In addition, CD109 silencing completely inhibited the growth of the MES-like BT13 tumors that consist of a large primary tumor (Figure 5, B, F, H, and J) as well as the growth of the PN-like BT18 and ZH305 models that grow very diffusively (Figure 5, C, D, and K–N).

Further characterization of the CD109-silenced MES-like xenografts revealed a significant reduction in the number of Ki-67–positive proliferating tumor cells and OLIG2-positive GSCs (Figure 5, O–R; and Supplemental Figure 5, F–I). Western blot analysis of whole tumor extracts demonstrated decreased expression of the human-specific GSC markers SOX2 and OLIG2 and reduced levels of p-STAT3 compared with the controls (Figure 5, S and T). None of the studied markers were detected in the brains of mice implanted with the CD109-silenced BT13 cells (Figure 5, Q, R, and T; and Supplemental Figure 5, H and I), confirming the complete abolishment of tumor initiation/formation. Expression of PDGFR-α and NG2 in tumor cells associates with the OPC-like cells (39). These progenitor cells were detected in the immediate vicinity of tumor blood vessels in the xenografts derived from the BT12 control cells (Figure 5, U and V). In the CD109-silenced xenografts, the number of PDGFR-α–positive and NG2-positive cells was significantly decreased (Figure 5, W and X), suggesting an important loss of the GSCs in the perivascular niche.

### CD109 orchestrates the tumor stroma establishment in vivo.

**Figure 6:**
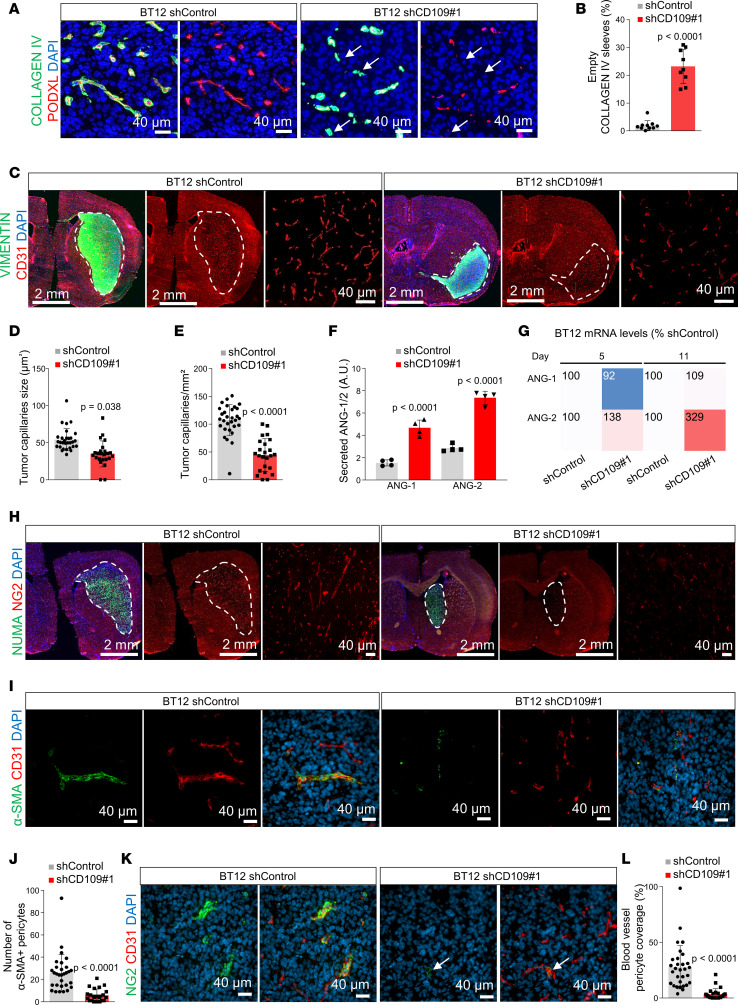
CD109 silencing modifies glioblastoma stroma. (**A**) Representative micrographs of BT12 xenografts immunostained with anti–collagen IV (green) and anti-podocalyxin (red). Nuclei were counterstained with DAPI (blue). Scale bar: 40 μm. Arrows indicate empty basement membrane sleeves devoid of endothelium. (**B**) Quantification of collagen IV^+^/PODXL^–^ empty sleeves in control and CD109-silenced BT12 xenografts (*n* = 10). (**C**) Representative micrographs of BT12 xenografts immunostained with anti-human vimentin (green) and anti-mouse CD31 (red). Nuclei were counterstained with DAPI (blue). Scale bars: 2 mm or 40 μm as indicated. Quantification of the average tumor blood vessel size (**D**) and density (**E**) in control and CD109-silenced BT12 xenografts (*n* = 10). (**F**) Quantification of the secreted ANG1 and ANG2 levels from the cell culture supernatants of CD109-silenced and nontargeted control BT12 GSCs (*n* = 2). (**G**) Expression of *ANGPT1* and *ANGPT2* mRNA levels determined by total RNA-Seq in CD109-silenced and nontargeted BT12 GSCs at the indicated time points. (**H**) Representative micrographs of BT12 xenografts immunostained with anti-human NUMA (green) and anti-NG2 (red). Nuclei were counterstained with DAPI (blue). Scale bars: 2 mm or 40 μm as indicated. (**I**) Representative micrographs of BT12 xenografts immunostained with anti–α-SMA (green) and anti-CD31 (red). Nuclei were counterstained with DAPI (blue). Scale bar: 40 μm. (**J**) Quantification of the α-SMA^+^ tumor pericytes in control and CD109-silenced BT12 xenografts (*n* = 10). (**K**) Representative micrographs of BT12 xenografts immunostained with anti-NG2 (green) and anti-CD31 (red). Nuclei were counterstained with DAPI (blue). Scale bar: 40 μm. (**L**) Quantification of the pericyte coverage in control and CD109-silenced BT12 xenografts (*n* = 10). *P* values were calculated by using the unpaired 2-tailed *t* test except for **F** and **G**, where a *t* test with Mann-Whitney post hoc test was used.

Beyond fueling oxygen and nutrients to support tumor growth, tumor blood vessels also shelter and nurture GSCs in perivascular niches promoting tumor resistance (40). Following CD109 silencing, vascular niches were less abundant in patient-derived xenografts. Costaining of the tumor blood vessels with type IV collagen revealed a significant increase in the collagen IV–positive “empty sleeves” devoid of endothelial marker in the CD109-silenced xenografts compared with controls (Figure 6, A and B). Further analysis confirmed a significant reduction in the blood vessel size and density in CD109-silenced xenografts (Figure 6, C–E).

Blood vessel growth and structural stability are regulated by angiogenic factors released by glioblastoma cells (40). We analyzed secreted factors in the culture media of the control and CD109-silenced GSCs and detected increased secretion of the proinflammatory cytokines, including Fas ligand, IL-1β, and TNF-α, following silencing (Supplemental Figure 6, A and B). Furthermore, CD109-silenced GSCs secreted considerably high levels of angiopoietin-2 (ANG2) compared with controls (Figure 6F and Supplemental Figure 6, A and B). Analysis of the angiopoietin-1 (*ANGPT1*) and ANG2 (*ANGPT2*) mRNA levels revealed a significant increase in the *ANGPT2* transcript levels, which in turn increased the ANG2/ANG1 ratio (Figure 6G and Supplemental Figure 6, C–E). ANG2 promotes endothelial destabilization due to its potent repulsive signals to pericytes (41), which led us to investigate the pericyte distribution in tumor blood vessels in the control and CD109-silenced BT12 xenografts. Immunostaining with pericyte markers α–smooth muscle actin (α-SMA) and NG2 (Figure 6, H–J) and PDGFR-β and NG2 (Figure 6, K and L; and Supplemental Figure 6, F and G) revealed a significant decrease in the pericyte coverage of CD109-silenced xenografts compared with controls.

## Discussion

Glioblastoma remains a clinical challenge particularly due to subpopulations of tumorigenic and therapeutically resistant GSCs that fuel tumor initiation and drive cellular heterogeneity, increasing therapeutic resistance. In this study, we report the discovery of CD109-GP130 interaction that promotes activation of the IL-6/STAT3 signaling pathway to maintain the stemness and tumorigenicity of GSCs and promote chemoresistance. Using a large cohort of clinical glioma samples and patient-derived GSCs, we established the association between CD109 and STAT3 phosphorylation. We studied the signaling mechanism further and observed that CD109 physically interacts with the GP130. Interestingly, loss of CD109 led to reduced STAT3 activation and stemness of the GSCs as well as substantially impaired tumorigenicity in vivo. Importantly, loss of CD109 or pharmacologic targeting of STAT3 sensitized the GSCs to chemotherapy. Our data indicate that therapeutic targeting of CD109/STAT3 axis in combination with chemotherapy provides a relevant therapeutic approach to increase the effectiveness of glioblastoma treatment.

Association between CD109 and STAT3 has appeared context dependently in previously reports. CD109 increased the levels of p-STAT3 in keratinocytes (42) and promoted the formation of lung adenocarcinoma metastasis via JAK/STAT3 pathway (43) whereas in glioblastomas such association has remained unexplored. In a recent study using a PDGF-β–driven genetically engineered mouse model of glioma, no association between STAT3 and CD109 was observed (20). However, the association was not studied in the clinical glioblastoma samples. Based on our findings, CD109-STAT3 association is a characteristic trait of glioblastomas. Establishment of a study model that would recapitulate human glioblastoma in laboratory animals has been challenging with certain limitations of the genetically engineered mouse models (44).

CD109 has been suggested to associate with the MES subtype and poor survival in TCGA GBM database (24). In accordance with these previous observations, we confirmed a strong association of CD109 with the MES subtype of glioblastoma across the independent data sets and poor survival in patients with gliomas. Furthermore, mRNA expression levels of CD109 and components of the STAT3 pathway were enriched in the MES subtype cell lines compared with the PN and CL subtype cell lines. Globally, loss of CD109 reduced the levels of p-STAT3 across all the studied GSCs regardless of the subtype.

We identified a potentially novel signaling mechanism in GSCs where CD109 physically interacts with GP130 to promote activation of IL-6/STAT3 pathway. Interestingly, soluble recombinant CD109 was sufficient to induce the interaction, indicating that CD109 is capable of mediating paracrine signaling effects (21).

Stem cells are multipotent with high adaptation potential, which creates a selective advantage to respond to therapies compared with differentiated cells having lower adaptation potential due to more restricted transcriptional programs (45). As an example of high cellular plasticity and drug resistance of glioblastoma cells, TMZ has been shown to induce a phenotypic shift of non-GSCs to a GSC state (11). Interestingly, we demonstrated that loss of CD109 in GSCs caused a selective pressure and changed the cell identity toward the more differentiated AC-like cells, accompanied with partial cell apoptosis and reduced tumorigenicity. Importantly, targeting CD109 or inhibiting STAT3 activity significantly sensitized the cells to TMZ (Figure 4I), supporting a model where targeting the CD109/STAT3 axis blocks the dedifferentiation of the cells, thereby enabling an opportunity for the TMZ treatment. This highlights the importance of the CD109/STAT3 axis in the maintenance of stemness and cellular plasticity. The importance of the CD109 expression in glioblastomas and treatment response has been recently suggested by Minata and colleagues (24) in the context of radiotherapy. Our study widens this view to the sensitization to chemotherapy underlying the clinically relevant role of CD109.

Although STAT3 is often hyperactivated in cancer, it is essential for normal development and physiology. In the developing nervous system, activation of JAK/STAT3 pathway promotes differentiation of neural stem and progenitor cells into astrocytes (46, 47). In embryonic stem cells, STAT3 promotes self-renewal and maintains pluripotency (48, 49). Furthermore, knockout of *STAT3* leads to embryonic lethality (31). Following its physiologic function in brain development and differentiation, our findings indicate that the STAT3 pathway is hijacked by glioblastoma cells and used as a switch for the GSCs to maintain their plasticity, which is fundamental to therapeutic resistance, tumor progression, and relapse. Despite major efforts, targeting STAT3 in tumor cells has proved challenging likely due to its cytosolic localization and lack of enzymatic activity (50).

CD109 is expressed at the plasma membrane and opens an alternative therapeutic window to target STAT3: (a) CD109 expression is highly upregulated in glioblastomas compared with the lower grade gliomas or normal brain, (b) high CD109 expression predicts poor survival and could therefore be used as a biomarker to predict poor treatment response and shorter survival, (c) CD109 expression is enriched in the GSCs compared with non-GSCs, increasing the selectivity of drug targeting with potentially fewer side effects to normal cells, (d) *CD109*-knockout mice are viable (15), and (e) perivascular localization of GSCs expressing CD109 would potentially facilitate the accessibility for molecules aiming at blocking the CD109/STAT3 axis to reprogram GSCs into a drug-sensitive state (45).

In conclusion, our study has uncovered an important signaling mechanism for the maintenance of stemness, plasticity, and tumorigenicity of GSCs and suggests that targeting the CD109/STAT3 axis provides a tool to eliminate the chemoresistant GSCs in the tumors to improve treatment efficacy.

## Methods

### Antibodies and reagents.

Western blotting and co-IP: monoclonal mouse CD109 (sc-271085, Santa Cruz Biotechnology) and GP130 (sc-376280, Santa Cruz Biotechnology), polyclonal goat SOX2 (AF2018, R&D Systems, Bio-Techne), OLIG2 (AF2418, R&D Systems, Bio-Techne), GP130 (AF228, R&D Systems, Bio-Techne), monoclonal mouse β-tubulin (556321, BD Biosciences), polyclonal rabbit p-STAT3 (Tyr705) (9131, Cell Signaling Technology), and monoclonal mouse STAT3 (9139, Cell Signaling Technology). Immunofluorescence: polyclonal sheep CD109 (AF4385, R&D Systems, Bio-Techne); monoclonal rabbit cleaved caspase-3 (Asp175) (9664, Cell Signaling Technology); phalloidin-TRITC, F-actin (P1951, MilliporeSigma); monoclonal mouse anti-human vimentin Cy3 conjugate (C9080, MilliporeSigma); monoclonal rat anti-mouse podocalyxin (MAB1556, R&D Systems, Bio-Techne) and CD31 (553370, BD Biosciences); monoclonal mouse anti-human nuclear marker (NUMA) Cy3 conjugate (MAB1281C3, MilliporeSigma); polyclonal rabbit NG2 (AB5320, MilliporeSigma) and anti-mouse collagen type IV (AB756P, MilliporeSigma); monoclonal mouse α-SMA Cy3 conjugate (C6198, MilliporeSigma); monoclonal rabbit anti-human lamin A/C (ab108595, Abcam); polyclonal goat IBA-1 (ab107159, Abcam); polyclonal goat anti-human PDGFR-α (AF-307 R&D Systems, Bio-Techne); polyclonal rabbit anti-mouse PDGFR-β (sc-436, Santa Cruz Biotechnology). Immunohistochemistry: polyclonal sheep CD109 antibody and polyclonal goat OLIG2 antibodies (AF-4385 and AF-2418, R&D Systems, Bio-Techne), monoclonal mouse Ki-67 (M7240, Dako). PLA: monoclonal mouse CD109 (MAB4385, R&D Systems, Bio-Techne), polyclonal goat GP130 (AF-228, R&D Systems, Bio-Techne), and monoclonal mouse 25kDa CD109 (11H3) (10381, Immuno-Biological Laboratories). Reagents: Recombinant human IL-6 (200-06, PeproTech) was reconstituted at 100 μg/mL in sterile water, and recombinant human CD109 (4385-CD, R&D Systems, Bio-Techne) was reconstituted at 100 μg/mL in sterile PBS. TMZ (HY-17364, MedChemExpress) and STAT3 inhibitor V, Stattic (573099, MilliporeSigma), were both reconstituted in DMSO.

### Clinical patient material and immunohistochemical analysis.

Clinical patient material was obtained from surgically operated patients at the Tampere University Hospital during 1983–2001. Tumors were graded and classified by a neuropathologist according to the WHO 2007 criteria (51). The study material consisted of 385 human astrocytoma samples: grade I *n* = 39, grade II *n* = 50, grade III *n* = 24, and grade IV *n* = 272 of which 265 cases were primary tumors and 120 recurrences.

For immunohistological analysis, 5 μm thick sections were deparaffinized and antigen was retrieved in Tris-HCl (pH 8.5) using a Lab Vision PT-module (Thermo Fisher Scientific) for 20 minutes at 98°C. Immunostaining was performed using Lab Vision Autostainer 480 (Thermo Fisher Scientific) and HRP/DAB detection system. Dako REAL antibody diluent served as a blocking reagent. Slides were incubated with primary CD109 antibody (R&D Systems, Bio-Techne; 1:400) diluted in the blocking buffer for 1 hour followed by 30 minutes’ incubation with HRP-conjugated secondary antibody (Dako). Staining was visualized using the Dako REAL DAB+ substrate, and the slides were counterstained with Mayer’s hematoxylin (S3309, Dako).

Intensity of CD109 immunostaining was manually scored in tumor cells on a 0–3 scale (0 = negative; 1 = weak; 2 = moderate; 3 = strong) blinded to the clinical data. Immunohistochemical staining for Ki-67 and p53 has been previously described (52). *EGFR* amplification was detected using chromogenic in situ hybridization (53). EGFR and p-STAT3 immunohistochemical staining have been previously described (54). IDH1-R132H mutation was detected with a point mutation–specific antibody and has been previously described (55). For Kaplan-Meier survival analysis, data were stratified based on the median expression of CD109 as CD109^hi^ (above median) or CD109^lo^ (below median).

### Cell culture.

Isolation of the patient-derived GSCs (BT3, BT3 CD133^+^, BT11, BT12, BT13, BT18, and BT27) has been previously described (27). Patient-derived glioma cell lines were maintained in serum-free DMEM/F12 medium supplemented with 1× B27 (both from Gibco, Thermo Fisher Scientific), 2 mM l-glutamine, 100 U/mL penicillin, 100 μg/mL streptomycin, 15 mM HEPES (all from Lonza), 0.02 μg/mL human EGF, and 0.01 μg/mL human FGF-basic (both from PeproTech). ZH305 and S24 GSCs (gift from Michael Weller, Department of Neurology, University Hospital and University of Zurich, Zurich, Switzerland) were maintained in serum-free Neurobasal-A medium (Gibco, Thermo Fisher Scientific) supplemented with 1× B27 supplement without vitamin A, 2 mM l-glutamine, 100 U/mL penicillin, 100 μg/mL streptomycin, 0.02 μg/mL EGF, and 0.02 μg/mL FGF-basic (27). Human 293FT cells (ATCC) were maintained in DMEM with 4.5 g/L glucose medium (Lonza) supplemented with 10% FBS, 2 mM l-glutamine, 100 U/mL penicillin, and 100 μg/mL streptomycin. H2 cell line, a giant cell glioblastoma (gift from Seppo Meri, Department of Bacteriology and Immunology, Translational Immunology Research Program, University of Helsinki, Helsinki, Finland), were maintained in RPMI 1640 medium (Lonza) supplemented with 10% FBS, 2 mM l-glutamine, 100 U/mL penicillin, and 100 μg/mL streptomycin (56).

### Lentiviral-shRNA vectors.

The following shRNA constructs from the RNAi Consortium (TRC) library (Broad Institute of MIT and Harvard) in pLKO.1 lentiviral vector were used to silence either CD109 expression: human sh*CD109*#1 (TRCN0000073649) and human sh*CD109*#2 (TRCN0000073648) or gp130 expression: human sh*IL6ST*#1 (TRCN0000058287) and human sh*IL6ST*#2 (TRCN0000058285). Complete targeting sequences were as follows (5′–3′): sh*CD109*#1, GCCGATCCTTACATAGATATT; sh*CD109*#2, GCAGTACATTTACTGTGCTTT; sh*IL6ST*#1, CGGCCAGAAGATCTACAATTA; and sh*IL6ST*#2, CCCATACTCAAGGCTACAGAA. Lentivirus was produced by transfecting shRNA constructs or nontargeting control vector (shControl) together with CMVg and CMV delta8.9 packaging plasmids (both from Addgene) and Fugene 6 transfection reagent (Promega) into 293FT cells. Silencing efficiencies of different shRNA constructs were confirmed using Western blotting and/or qRT-PCR.

### Western blotting.

Cells were washed with ice-cold PBS and lysed in RIPA buffer (150 mM Tris-HCl pH 7.4, 150 mM NaCl, 1% sodium deoxycholate, 1% SDS, 2% Octyl-β-d-glucopyranoside, 0.5 mM DTT) containing protease and phosphatase inhibitor cocktails (both from Roche). Samples were lysed on ice for 30 minutes, sonicated, and microcentrifuged at 18,000 RCF for 20 minutes at 4°C. Protein concentrations were determined using the Pierce BCA Protein Assay Kit (Thermo Fisher Scientific) according to the manufacturer’s instructions. Samples were boiled in reducing Laemmli sample buffer, and 10 μg of protein per lane was separated on Tris-Glycine Mini Gels (Invitrogen, Thermo Fisher Scientific). Proteins were transferred onto the PVDF membrane using the Transblot Turbo transfer system (Bio-Rad). Membranes were blocked in 5% nonfat dried milk in 0.1% TBS with Tween (TBS-T) for 1 hour at room temperature and then incubated with primary antibodies overnight at 4°C. After washes with 0.1% TBS-T, membranes were incubated with appropriate HRP-conjugated secondary antibodies (Dako). Finally, the signal was developed with Supersignal West Pico ECL substrate (Thermo Fisher Scientific) or Clarity Western ECL substrate (Bio-Rad) and exposed to Super RX-N FUJIFILM Medical X-ray Film. See complete unedited blots in the supplemental material.

### Coimmunoprecipitation.

Cells were washed with ice-cold PBS and carefully lysed in IP buffer (20 mM Tris-HCl pH 7.5, 150 mM NaCl, 1% Triton X-100, 1 mM EDTA, 1 mM EGTA, 1 mM β-Glycerophosphate, 2.5 mM sodium pyrophosphate, 1 μg/mL leupeptin) and protease and phosphatase inhibitor cocktails. Lysates were incubated on ice for 5 minutes and then microcentrifuged at 14,000*g* for 10 minutes at 4°C. Protein concentrations were determined as previously described. Cell lysates were precleared by incubating with prewashed Dynabeads Protein G (Thermo Fisher Scientific) for 30 minutes at room temperature at rotation. A total of 700 μg of total protein was immunoprecipitated together with 7.5 μg of gp130 antibody or appropriate isotype control antibody overnight at 4°C with rotation. Immunocomplexes were incubated with prewashed Dynabeads Protein G for 1 hour 30 minutes at room temperature. Immunocomplexes bound to the magnetic beads were washed 5 times with ice-cold IP lysis buffer and eluted by boiling in reducing 4× Laemmli Sample Buffer (Bio-Rad). Samples were subjected to SDS-PAGE followed by Western blotting as described previously. The experiments were repeated twice.

### Proximity ligation assay.

PLA was performed using the Duolink In Situ Red Starter Kit Mouse/Goat (MilliporeSigma) according to the manufacturer’s protocol. Briefly, GSCs were plated as single cells on Lab-Tek II chamber slides (Nunc, Thermo Fisher Scientific) coated with Laminin (1 μg/cm^2^; Invitrogen, Thermo Fisher Scientific) and incubated overnight at 37°C. Cells were treated with and without recombinant human CD109 (500 ng/mL) for 4 hours, as indicated. Slides were fixed with 4% paraformaldehyde (PFA) for 10 minutes at room temperature, blocked for 1 hour at room temperature, and incubated with primary antibodies CD109 (mouse), CD109 11H3 (mouse), and gp130 (goat) diluted 1:200 in antibody diluent overnight at 4°C. PLA probe incubation, ligation, and amplification steps were performed according to the protocol. Finally, slides were mounted in mounting medium with DAPI and imaged using a confocal microscope (Zeiss LSM880). The experiments were repeated twice.

### Quantitative real-time PCR.

RNA was isolated using the Nucleospin RNA II kit (Macherey-Nagel), and cDNA was synthesized using the iScript cDNA synthesis kit (Bio-Rad) according to the manufacturer’s instructions. Quantitative PCR was performed using the CFX96 real-time PCR detection system (Bio-Rad) and SYBR FAST qPCR Master Mix (Kapa Biosystems). Gene expression was normalized to the expression of the human ADP-ribosylation factor 1 (*ARF1*) housekeeping gene using the comparative Ct method. Fold changes were calculated relative to control.

Complete primer sequences were as follows (5′–3′): *CD109*, fwd: GTCCACATGTCCGAAAGCAT, rev: AAACCAGTAGCCACCCAAGAA, *SOX2*, fwd: CACACTGCCCCTCTCAC, rev: TCCATGCTGTTTCTTACTCTCC, *SOX4*, fwd: GGTCTCTAGTTCTTGCACGCT, rev: CTGCAAGAAGGGAGCTGGTAA, *NANOG*, fwd: GAAATACCTCAGCCTCCAGC, rev: GCGTCACACCATTGCTATTC, *OLIG2*, fwd: AGCTCCTCAAATCGCATCC, rev: ATAGTCGTCGCAGCTTTCG, *POU5F1*, fwd: CGAGAAGGATGTGGTCCGAG, rev: TGTGCATAGTCGCTGCTTGA, *PROM1*, fwd: TGGATGCAGAACTTGACAACGT, rev: ATACCTGCTACGACAGTCGTGGT, *NES1*, fwd: GGCTGCGGGCTACTGAAA, rev: CTGAGCGATCTGGCTCTGTAG, *MAP2*, fwd: TGCCTCAGAACAGACTGTCAC, rev: GGCTCTTGGTTACTCCGTCA, *GALC*, fwd: GGATCTTACAGGGTTACAGGTGA, rev: TGCTGTAACTTCAACACGTCCT, *GFAP*, fwd: GCAACATGCATGAAGCCGAA, rev: GGGACTCGTTCGTGCCG, *IL6RA*, fwd: CTGGAAACTATTCATGCTACC, rev: GACTGTTCTGAAACTTCCTC, *IL6ST*, fwd: ACCAGTACACTATCTTGGTC, rev: TTTAATGTCCACTAGGAGGG, *ANG1*, fwd: AACATGGGCAATGTGCCTACACTT, rev: CATTCTGCTGTATCTGGGCCATCT, *ANG2*, fwd: CAGATTTTGGACCAGACCAGTGA, rev: TCAATGATGGAATTTTGCTTGGA, *ALDOC*, fwd: CCAATACAAGAAGGATGGTG, rev: CAGGCAATATTTCAGGTTCC, *AQP4*, fwd: CATTGGATATATTGGGTTGGG, rev: TCAACATCTGGACAGAAGAC, *EGFR*, fwd: TCCAGTGGCGGGACATAGT, rev: TGGATCACACTTTTGGCAGC, *FGFR3*, fwd: CCTTTGTCCTTTTTCAGGAG, rev: CTAATAACATCGGAACCTGC, *SLC1A3*, fwd: CCCTTACAAAATCAGAAAAGTTG, rev: TTTTCTAGGAGGGTCTCTTC, *ARF1*, fwd: TCCCACACAGTGAAGCTGATG, rev: GACCACGATCCTCTACAAGC.

### Proliferation and cell viability assay.

For cell proliferation and viability assays, 5 × 10^3^ cells were plated in triplicates in 96-well plates in complete culture media with growth factors. At indicated time points, cells were treated with 10 μL of 3-(4,5-dimethylthiazol-2-yl)-2,5-diphenyltetrazolium bromide (MTT; 5 mg/mL in PBS) and incubated for 2 hours at 37°C. Cells were lysed in 10% SDS-10 mM HCl overnight, and absorbance was measured at 540 nm using a FLUOstar Omega microplate reader (BMG Labtech). The experiments were repeated 3 times.

### Drug sensitivity assays.

For TMZ sensitivity experiments, 3 different control and CD109-silenced GSCs were plated at 5 × 10^3^ cells/well in 96-well plates in complete culture media with growth factors and incubated for overnight. The following day, cells were treated with 250 μM of TMZ or DMSO for 4 days. TMZ treatment was repeated after 2 days of culture. MTT assay was performed as described above. The experiments were performed 3 times with 5 replicates.

For a combination therapy with Stattic and TMZ, 3 different lines of GSCs were plated at 5 × 10^3^ cells/well in 96-well plates in complete culture media with growth factors and treated with 1 μM of Stattic overnight. The following day, cells were treated with 250 μM of TMZ and incubated for 2 days. MTT assay was performed as described above. The experiments were performed 3 times with 5 replicates.

### Colony forming assay.

A total of 1 × 10^4^ control or CD109-silenced GSCs were suspended as single cells in a complete growth medium with the growth factors EGF and FGF-basic and mixed with semisolid methylcellulose stock solution (R&D Systems, Bio-Techne) to a final concentration of 1.3%. One milliliter of the mixture was added into a 35 mm plate in triplicates. Experiments were started 4 days after lentiviral transduction. Colonies were incubated for 3 weeks at 37°C containing 5% CO_2_. Colonies were imaged using Nikon Eclipse Ti-E inverted microscope and quantified using the CellProfiler cell image analysis software (http://cellprofiler.org/citations/). The experiments were repeated 3 times.

### Cytokine array.

Secretion of selected human cytokines and chemokines to the cell culture medium was determined using the proteome profiler human XL cytokine array kit (R&D Systems, Bio-Techne). A total of 500 μL of cell culture supernatant from control and CD109-silenced GSCs at day 5 was run on the array according to the manufacturer’s instructions. Signal was visualized using chemiluminescence detection and developed on x-ray film. The average pixel density of each analyte from duplicate spots was determined, and fold changes were calculated relative to control.

### 3D fibrin cultures.

Fibrinogen (6 mg/mL; Calbiochem) was dissolved in HBSS for 2 hours at 37°C. GSCs (3 × 10^3^) transduced with shControl or shCD109 were suspended in 15 μL of fibrinogen solution at room temperature. Then 15 μL of thrombin (4 U/mL) and aprotinin (400 μg/mL) in HBSS medium was added to the mixture. After suspending carefully, 30 μL fibrin drops were pipetted into 24-well plates and allowed to polymerize for 1 hour at 37°C. After polymerization, complete DMEM/F12 medium, growth factors (EGF and FGF), and aprotinin (100 μg/mL) were added to the 3D cultures. Fresh growth medium, growth factors, and aprotinin were supplied to the cultures twice weekly. The cell growth was followed by EVOS FL inverted epifluorescence microscope (Thermo Fisher Scientific). At indicated time points, the cultures were fixed with 4% PFA in PBS for 1 hour at room temperature, washed with PBS, and stored at 4°C for immunofluorescence stainings.

### ATP measurement.

Viable cells present in the 3D fibrin cultures were quantified based on the released ATP levels using CellTiter-Glo 3D Cell Viability Assay (Promega) according to the manufacturer’s instructions. Briefly, at indicated time points, cells were mixed with CellTiter-Glo 3D reagent in 96-well plates, vortexed, and then incubated for 25 minutes at room temperature. Luminescence was measured using the FLUOstar Omega Microplate Reader (BMG Labtech).

### Immunofluorescence of the cell death.

In order to attach the suspension cells, glass coverslips were first coated with poly-d-lysine (MilliporeSigma) according to the manufacturer′s instructions. Cells were cultured for 24 hours after which they were fixed with 4% PFA and permeabilized with 0.3% Triton X-100 in PBS. The cells were blocked (10% FBS, 0.03% Triton X-100–PBS) and incubated with the anti–cleaved caspase-3 and CD109 primary antibodies (1:200) and fluorescently labeled secondary antibodies (Alexa Fluor, Life Technologies, Thermo Fisher Scientific). Nuclei were visualized with DAPI (VECTASHIELD, Vector Laboratories).

### TUNEL staining.

Apoptotic and necrotic cells with DNA strand breaks were labeled using the In Situ Cell Death Detection Kit, TMR red (Roche), according to the manufacturer’s recommendations. Briefly, in order to attach the suspension cells, glass coverslips were first coated with poly-d-lysine according to the manufacturer′s instructions. Cells were cultured for 24 hours after which they were fixed with 4% PFA. Cells were then treated with proteinase K (15 μg/mL in 10 mM Tris-HCl, pH 7.5) at 37°C for 25 minutes and incubated with the TUNEL reaction mixture at 37°C for 1 hour. Finally, coverslips were stained with DAPI and mounted with Mowiol (MilliporeSigma). Entire coverslip scanned images were generated using 3DHISTECH Pannoramic 250 FLASH II digital slide scanner at the Genome Biology Unit (University of Helsinki and Biocenter Finland).

### Immunofluorescence staining of the 3D fibrin cultures.

The 3D fibrin cultures were briefly postfixed in ice-cold acetone-methanol (1:1), washed with PBS, and blocked in 15% FBS and 0.3% Triton X-100 in PBS for 2 hours at room temperature. The 3D fibrin cultures were then incubated in primary antibodies, diluted in the blocking buffer, overnight at 4°C. After several long washes, first with 0.45% Triton X-100 in PBS and then with PBS, the 3D fibrin cultures were incubated with the appropriate fluorescent secondary antibodies for 4 hours at room temperature. Finally, the 3D fibrin cultures were washed with PBS and mounted with VECTASHIELD mounting medium with DAPI. The samples were imaged using a Zeiss AxioImager.Z1 upright epifluorescence microscope equipped with Apotome and Hamamatsu Orca R2 1.3-megapixel monochrome charge-coupled device camera.

### Intracranial orthotopic tumor xenografts.

Animal experiments were approved by the Committee for Animal Experiments of the District of Southern Finland (ESAVI/6285/04.10.07/2014 and ESAVI/403/2019). Intracranial implantation of patient-derived gliospheres was performed as described (27). Briefly, 6-week-old Rj:NMRI-Foxn1nu/Foxn1nu (Janvier Labs) mice were placed under 2.5% isoflurane anesthesia on a stereotaxic injector and intracranially engrafted with 1 × 10^5^ cells (in 5 μL PBS) obtained from dissociated gliospheres at the following distance from the bregma: –1 mm anteroposterior, +2 mm right, +2.5 mm depth. Postoperative analgesia (temgesic) was locally administered for 2 days. At the endpoint of the experiment (>25 days of tumor growth), animals were euthanized by cervical dislocation, and brains were snap-frozen in −50°C isopentane (Honeywell) until tissue processing.

### Immunofluorescence staining of tumor tissue xenografts.

Snap-frozen xenografted brains were cut, from the frontal to the posterior part of the diencephalon, using a cryotome (Leica CM1950) into 9 μm thick coronal sections serried on microscope slides (Supplemental Figure 4A). Before immunofluorescence/histochemistry, brain sections were fixed in 4% PFA and blocked with 5% FBS and 0.03% Triton X-100 (MilliporeSigma). Whole slides were scanned using a slide scanner (3DHistec). Tumor volumes were calculated using the histological coordinates defined by the brain section series (Supplemental Figure 4A). If antibody species were incompatible for simultaneous costaining, verification was performed using consecutive microscope slides, on 9 μm distant consecutive brain sections (Figure 4, A and B). Brain structures (e.g., blood vessels, hippocampus, etc.) were used as spatial references. Histology quantification (e.g., tumor cell invasion, blood vessel density, pericyte coverage, etc.) was performed on 10 sections equally distributed along the entire tumor, for each sample.

### Microscope imaging.

For the light microscopy, the cells were visualized and imaged with an inverted epifluorescence microscope (Zeiss Axiovert 200) equipped with AxioVision software. Confocal images were taken with Zeiss LSM 780 and 880 equipped with appropriate lasers and equipped with Zeiss Zen Black software.

### Total RNA-Seq of patient-derived glioblastoma cells.

Total RNA was extracted from lysed cells or gliospheres using the RNeasy Mini Kit (74104, QIAGEN) according to the manufacturer’s instructions. After DNase treatment, the quality of the RNA preparations (0.5 μg) was controlled using a Tapestation (Agilent). Total RNA (0.5 μg) was treated with NEBNext rRNA Depletion Kit (Human/Mouse/Rat) (New England Biolabs [NEB] E6310) to remove the rRNA. First the samples were treated with rRNA removal solution after which the rRNA was removed, combining probe-hybridized samples and magnetic beads. The ribodepleted RNA was purified with the QIAGEN RNeasy MinElute Cleanup Kit. The absence of rRNA and the quantity of RNA were assessed by the Bioanalyzer (Agilent High Sensitivity DNA chip). NEBNext Ultra II Directional RNA Library Prep Kit for Illumina (NEB E7760, E7765) was used to generate cDNA libraries for next-generation sequencing. First, the rRNA-depleted sample (10 ng) was fragmented to generate inserts around 200 bp, then primed with random primers. The first-strand cDNA synthesis utilized actinomycin D, which inhibits the DNA polymerase activity of the reverse transcriptase, increasing strand specificity. In the second-strand cDNA synthesis dUTP-labeled oligonucleotides were incorporated to mark the second strand with uracils (U). The cDNA synthesis product was purified with the Agencourt AMPure XP beads. Next, the cDNA was end-repaired and adapter-ligated utilizing dA-tailing. The adapter-ligated DNA went through a bead-based size selection after which the final PCR enrichment took place. At this point, a unique index was provided to each sample to enable pooling of multiple samples (multiplexing) for sequencing. During the high-fidelity PCR, Uracil-specific Excision Reagent enzyme cuts away the uracil strand, preserving only the first strand. In addition, the loop adapter is cut open to enable the PCR. The amplified library was then purified using AMPure XP Beads. The library quality was assessed by the Bioanalyzer and library quantity by the Qubit (Invitrogen, Thermo Fisher Scientific). RNA-Seq was then performed on an Illumina NextSeq500 High Output sequencer (Functional Biology Unit, Finland).

### Data analyses of the total RNA-Seq.

The data discussed in this publication have been deposited in NCBI’s Gene Expression Omnibus (GEO) (57) and are accessible through GEO Series accession number GSE169418 (https://www.ncbi.nlm.nih.gov/geo/query/acc.cgi?acc=GSE169418).

Bcl2fastq2 Conversion Software was used to convert BCL files to FASTQ file format and demultiplex samples. Sequenced reads were trimmed for adapter sequence and masked for low-complexity or low-quality sequence using Trimmomatic. Parameters used::2:30:10 LEADING:3 TRAILING:3 SLIDINGWINDOW:4:15 MINLEN:36. Trimmed reads were mapped to GRCh38.p12 reference genome or GRCh38.p13 from GENCODE human release v.33 using STAR aligner (2.6.0c) with parameters --chimSegmentMin 12 --chimJunctionOverhangMin 12 --chimSegmentReadGapMax 3 --alignSJoverhangMin 8 --alignSJDBoverhangMin 1 --alignIntronMin 20 --alignIntronMax 1000000 --alignMatesGapMax 1000000 --alignSJstitchMismatchNmax 5 -1 5 5 --outFilterType BySJout --outFilterMismatchNmax 999 --outSAMattributes NH HI NM MD AS nM jM jI XS --outSAMtype BAM SortedByCoordinate --outSAMmapqUnique 60 --quantMode GeneCounts --twopassMode Basic. Counts per gene were calculated using the featureCounts software. Parameters used: -s 2 -T 20 -t exon -g gene_id -a gencode.v28.chr_patch_hapl_scaff.annotation.gtf. Differential expression analysis used the DESeq2 software in the R environment. Procedures included normalization of count values between samples using a geometric mean, estimation of sample-wise factors to correct for library size variability (for example), estimation of dispersion (i.e., variance, scatter) of gene-wise values between conditions, negative binomial linear model and Wald’s test to produce *P* values, removal of low-expression outlier (using Cook’s distance) results to optimize for *P* value adjustment, and finally, multiple-testing adjustment of *P* values with Benjamini-Hochberg procedure. The *z* score was determined as: reads of each sample – mean of all samples/SD. For the molecular subtype (35) and the phenotypic state profiling (10), the list of genes for the given signature was retrieved from their respective publications, and individual *z* scores were calculated among all samples.

### Statistics.

All statistics were computed using the GraphPad Prism 8 and IBM SPSS Statistics for Windows, version 22, software. Statistical significance between sample groups was determined using an unpaired 2-tailed *t* test or the nonparametric Mann-Whitney *U* test. For multiple comparisons, 1-way ANOVA with the Kruskal-Wallis test, or 2-way ANOVA with Sidak’s or Tukey’s multiple comparisons tests, were used. In the tumor microarray data set, the significance of associations was determined using the Pearson χ^2^ test. Survival analysis was performed using the Kaplan-Meier method and log-rank test. Data are presented as mean ± SD or ± SEM, and *P* < 0.05 was considered statistically significant. Statistical tests are specified in the figure legends. All images and graphs shown are representative of several experiments as indicated in the figure legends.

### Study approval.

The use of clinical patient material for tumor microarrays was approved by the Ethics Committee of Tampere University Hospital. The use of human glioma tissue biopsies was approved by the Ethics Committee of the Northern Savo Hospital District (53/2009; 1.8.2009-31.7.2019, Kuopio, Finland). All patients gave their written informed consent. All experiments involving animals were authorized by the National Animal Experimental Board in Finland (Helsinki, Finland).

## Author contributions

Conceptualization was done by PF, VLJ, and PL; methodology and visualization were designed by PF and VLJ; investigation was performed by PF, VLJ, KJG, JTR, HH, and EG; writing of the original draft was performed by PF; review and editing of the draft were performed by VLJ and PL; resources were provided by HH and PL; supervision was provided by VLJ, KJG, MN, KL, and PL; and funding acquisition and project administration were performed by PL.

## Supplementary Material

Supplemental data

## Figures and Tables

**Table 1:**
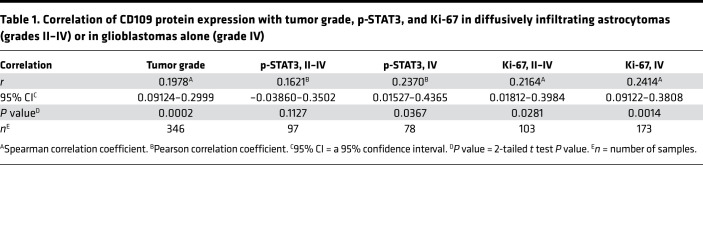
Correlation of CD109 protein expression with tumor grade, p-STAT3, and Ki-67 in diffusively infiltrating astrocytomas (grades II–IV) or in glioblastomas alone (grade IV)

## Version 1 05/10/2021

Electronic publication
