# Supplementary material for: CD109-GP130 interaction drives glioblastoma stem cell plasticity and chemoresistance through STAT3 activity
Source: JCI Insight. 2021 May 10;6(9):e141486. doi: 10.1172/jci.insight.141486 (PMC8262342; doi:10.1172/jci.insight.141486)
Supplement: Supplemental data [file jciinsight-6-141486-s008.pdf]

CD109 – GP130 interaction drives glioblastoma stem cell plasticity and chemoresistance through STAT3 activity

Pauliina Filppu[1], Jayendrakishore Tanjore Ramanathan[1], Kirsi Granberg[2,3], Erika Gucciardo[4], Hannu Haapasalo[5], Kaisa Lehti[4,6,7], Matti Nykter[2], Vadim Le Joncour[1*], and Pirjo Laakkonen[1,8*]

Supplemental figures and legends and supplementary tables

Supplemental Figure 1

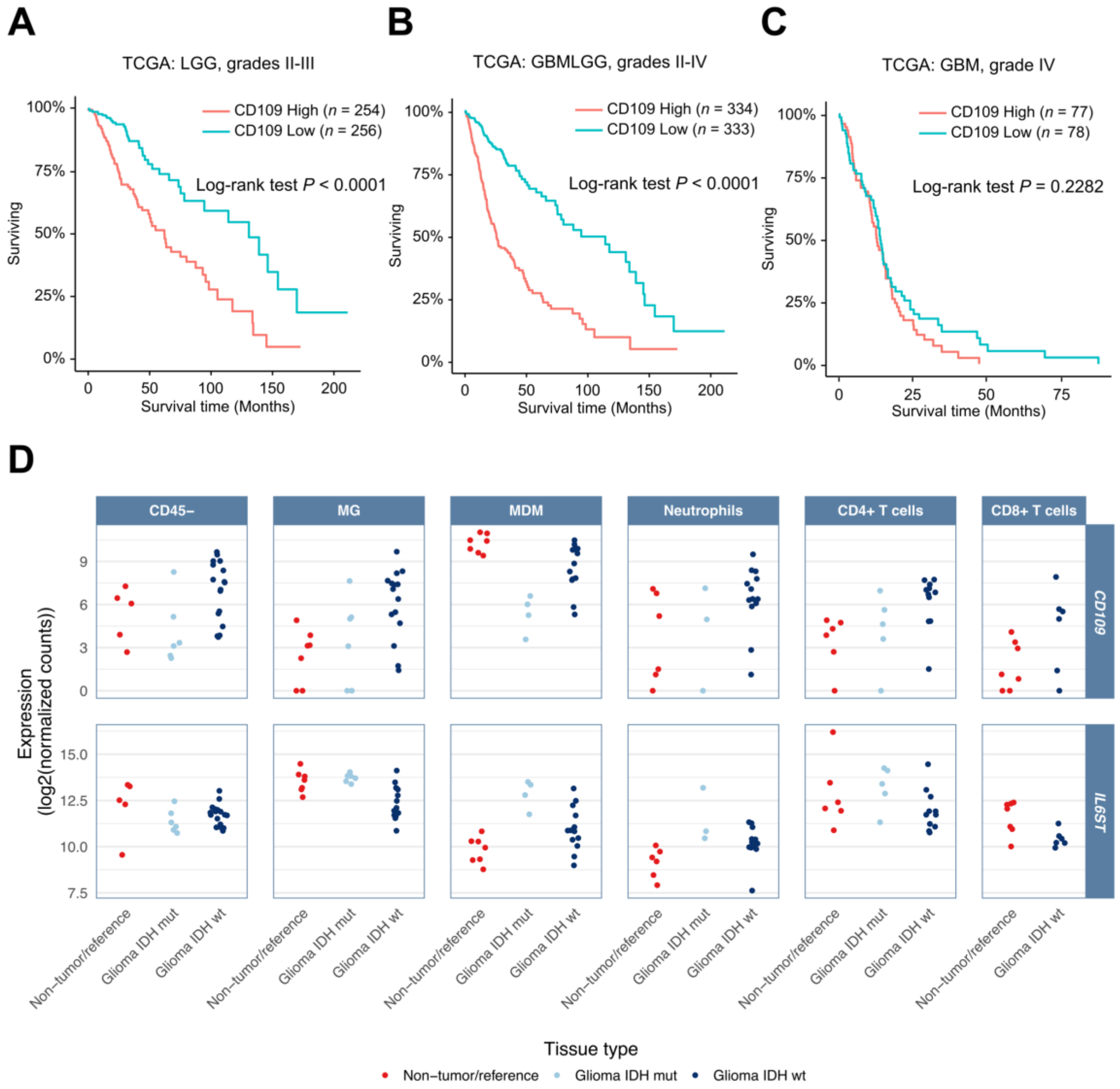

Supplemental Figure 1. CD109 expression associates with poor survival in gliomas. (**A-C**) Kaplan-Meier survival analyses based on CD109 expression. The median values were used as cutoffs. (**A**) The TCGA dataset of lower-grade gliomas (grades II-III), CD109 high (*n* = 254) red line and CD109 low (*n* = 256) blue line. ****P* < 0.001, log-rank test. (**B**) The TCGA dataset of glioblastoma and lower-grade gliomas (grades II-IV), CD109 high (*n* = 334) red line and CD109 low (*n* = 333) blue line. ****P* < 0.001, log-rank test. (**C**) The TCGA dataset of glioblastoma (grade IV), CD109 high (*n* = 77) red line and CD109 low (*n* = 78) blue line. (**D**) *CD109* and *IL6ST* mRNA level

expression across different immune cell types between non-tumor reference and glioma specimens in brain tumor immune microenvironment dataset.

Supplemental Figure 2

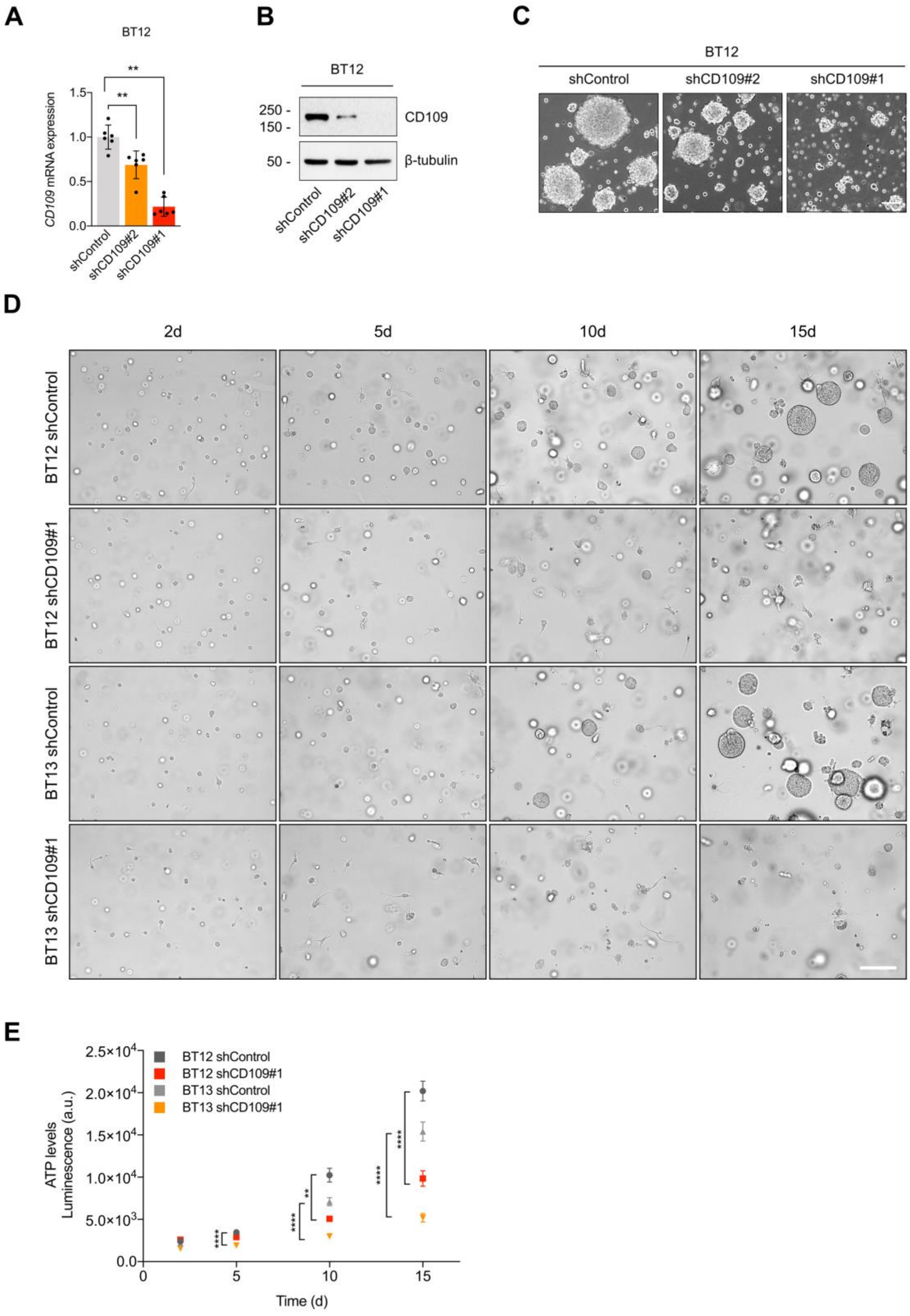

Supplemental Figure 2. CD109 silencing inhibits GSC growth. (**A**) qRT-PCR analysis of *CD109* mRNA levels in GSCs after CD109 silencing. Data are presented as mean ± SD. $^{**}P < 0.01$, non-

parametric Mann-Whitney *U* test. (**B**) Western blot analysis of CD109 expression in CD109-silenced and non-targeted control GSCs. β-tubulin served as a loading control. (**C**) Representative micrographs of CD109-silenced and non-targeted control GSCs in vitro at d11. CD109 silencing significantly decreased the size of gliospheres. Scale bar: 100 μm. (**D**) Representative micrographs of CD109-silenced and non-targeted control GSC in a 3D fibrin matrix at the indicated time points. Scale bar: 200 μm. (**E**) Cell viability of CD109-silenced and non-targeted control GSCs in 3D fibrin matrix at the indicated time points. Data are presented as mean ± SEM. $^{**}P < 0.01$; $^{****}P < 0.0001$, unpaired two-tailed t-test. Representative images of $n = 2$ (**C**) or $n = 3$ (**D**) independent experiments are shown. Data are from $n = 3$ (**A**, **B**, and **E**) independent experiments.

Supplemental Figure 3.

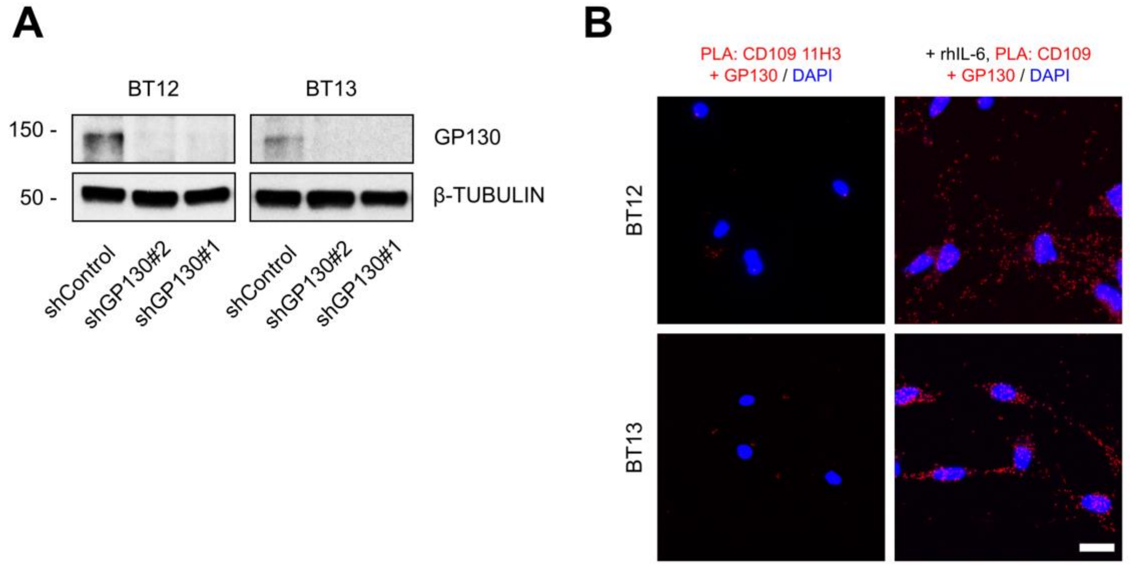

Supplemental Figure 3. CD109 physically interacts with gp130. (**A**) Western blot analysis of GP130 expression in control and GP130-silenced GSCs. β-tubulin served as a loading control. (**B**) Representative micrographs of PLA assay in GSCs using anti-CD109 11H3 (which detects the 25-kDa CD109 subunit) and anti-GP130 antibodies (left) and anti-CD109 and anti-GP130 antibodies in the presence of recombinant human IL-6 (50 ng/ml) (right). Red indicates specific interaction signal. Nuclei were counterstained with DAPI (blue). Scale bar: 20 μm. Data are from *n* = 2 (**B**) or *n* = 3 (**A**) independent experiments.

Supplemental Figure 4

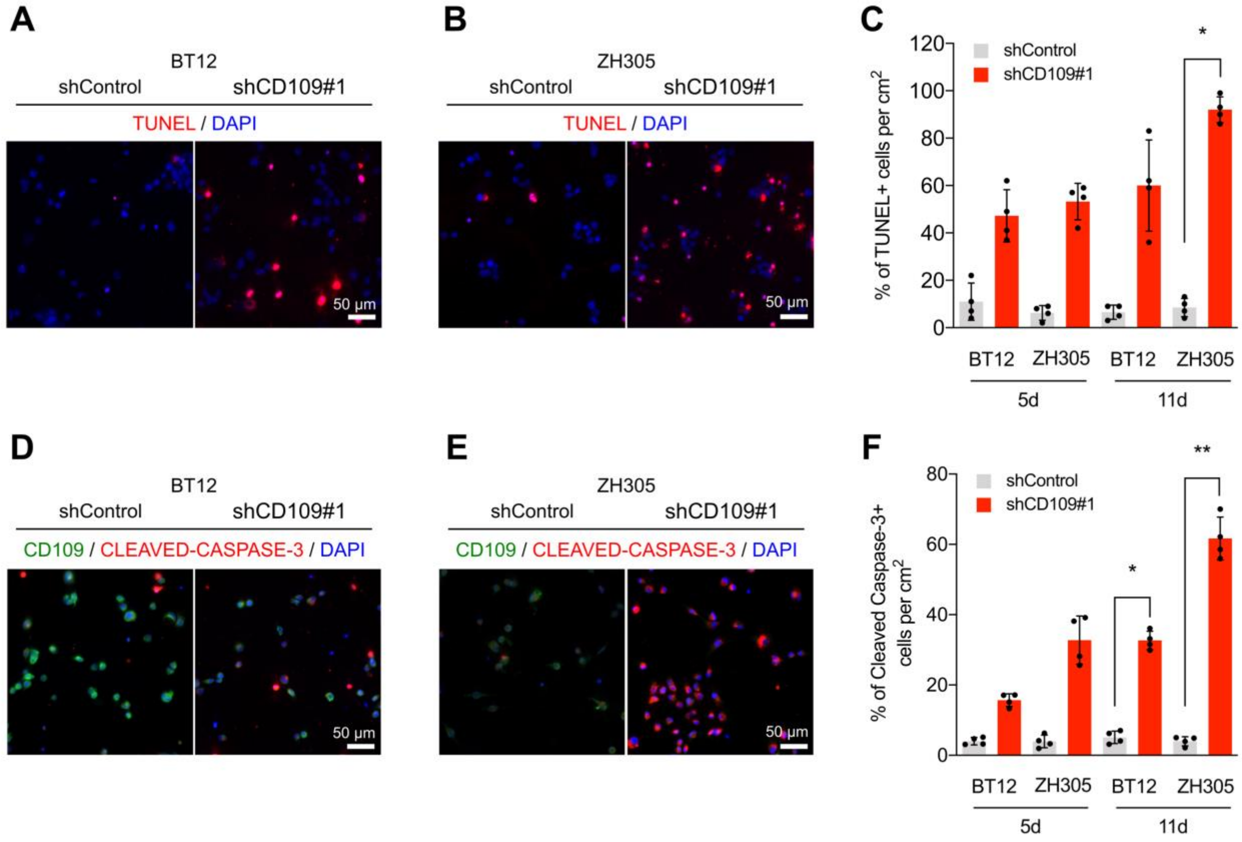

Supplemental Figure 4. CD109 silencing induces partial cell apoptosis. (**A** and **B**) Representative micrographs of TUNEL staining in CD109-silenced and non-targeted control GSCs. TUNEL signal is shown in red. Nuclei were counterstained with DAPI (blue). Scale bar: 50 µm. (**C**) Quantification of TUNEL+ cells at the indicated time points (n = 4). Data are presented as mean ± SD. *$P < 0.05$, one-way ANOVA with Kruskal-Wallis posthoc test. **(D** and **E)** Immunofluorescent staining of CD109 (green) and cleaved caspase-3 (red) in CD109-silenced and non-targeting control GSCs. Nuclei were counterstained with DAPI (blue). Scale bar: 50 µm. (**F**) Quantification of the cleaved caspase-3+ cells at the indicated time points (n = 4). Data are presented as mean ± SD. *$P < 0.05$; **$P < 0.01$, one-way ANOVA with Kruskal-Wallis posthoc test.

Supplemental Figure 5

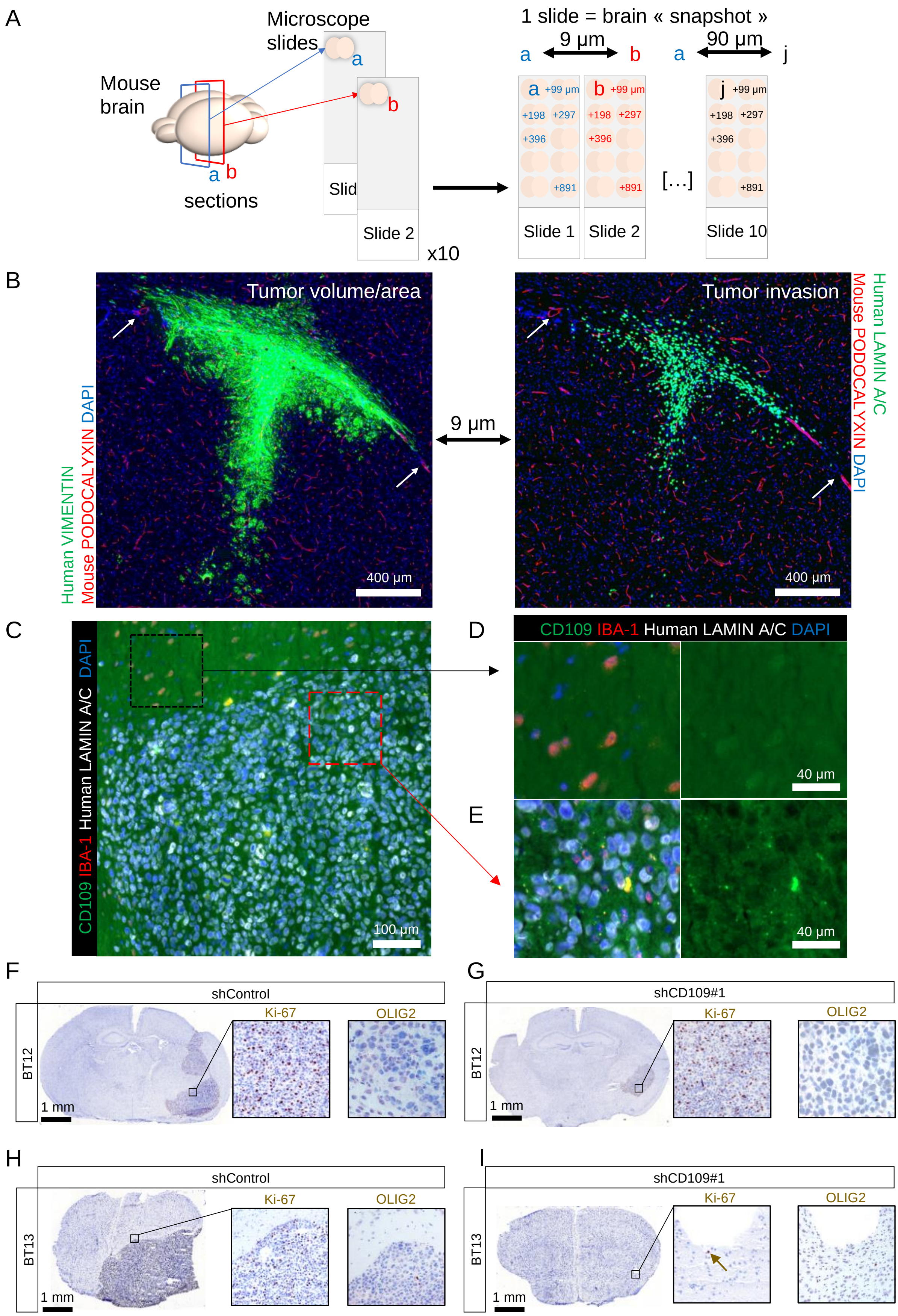

Supplemental Figure 5. Histological analysis of xenograft tumors. (**A**) Schematic representation of the brain "snapshots" technique. Cryosections (9 µm) of mice brains were collected from the frontal to posterior part of the xenografts and distributed on 10 to 20 microscope slides. On one slide, each section is 99 µm apart from the previous one. Following the same scheme, one microscope slide is 9 µm from the previous one. This allows to get a "snapshot" of the entire tumor on one slide, and close replicates for co-location studies when taking two consecutive slides. (**B**) Representative micrograph of anti-human vimentin immunostaining (green, left) to visualize the tumor cells in the mouse brain and to quantify the tumor area/volume and number of satellite tumors. Anti-human lamin A/C immunostaining (green, right) was used to quantify the single invasive tumor cells. Anti-mouse PODXL immunostaining (red) was used for quantification of the blood vessel density. Nuclei were counterstained with DAPI (blue). Scale bar: 400 µm. The precise location between consecutive sections was determined based on specific brain structures and blood vessels (arrows). (**C**) Representative micrograph of CD109 (green), microglial marker IBA-1 (red), and lamin A/C (white) immunostaining of BT12 xenografts. Nuclei were counterstained with DAPI (blue). Scale bar: 100 µm. (**D** and **E**) Higher magnification ROIs outlined in (**C**) of non-tumor brain stroma (**D**) and tumor tissue (**E**). Scale bar: 40 µm. (**F** and **G**) Representative micrographs of Ki-67 and OLIG2 IHC staining of control (**F**) and CD109-silenced (**G**) BT12 xenografts. Nuclei were counterstained with hematoxylin. Scale bar: 1 mm. (**H** and **I**) Representative micrographs of Ki-67 and OLIG2 IHC staining of control (**H**) and CD109-silenced (**I**) BT13 xenografts. Nuclei were counterstained with hematoxylin. Scale bar: 1 mm. The arrow indicates one single human cell found at the tumor implantation site.

Supplemental Figure 6

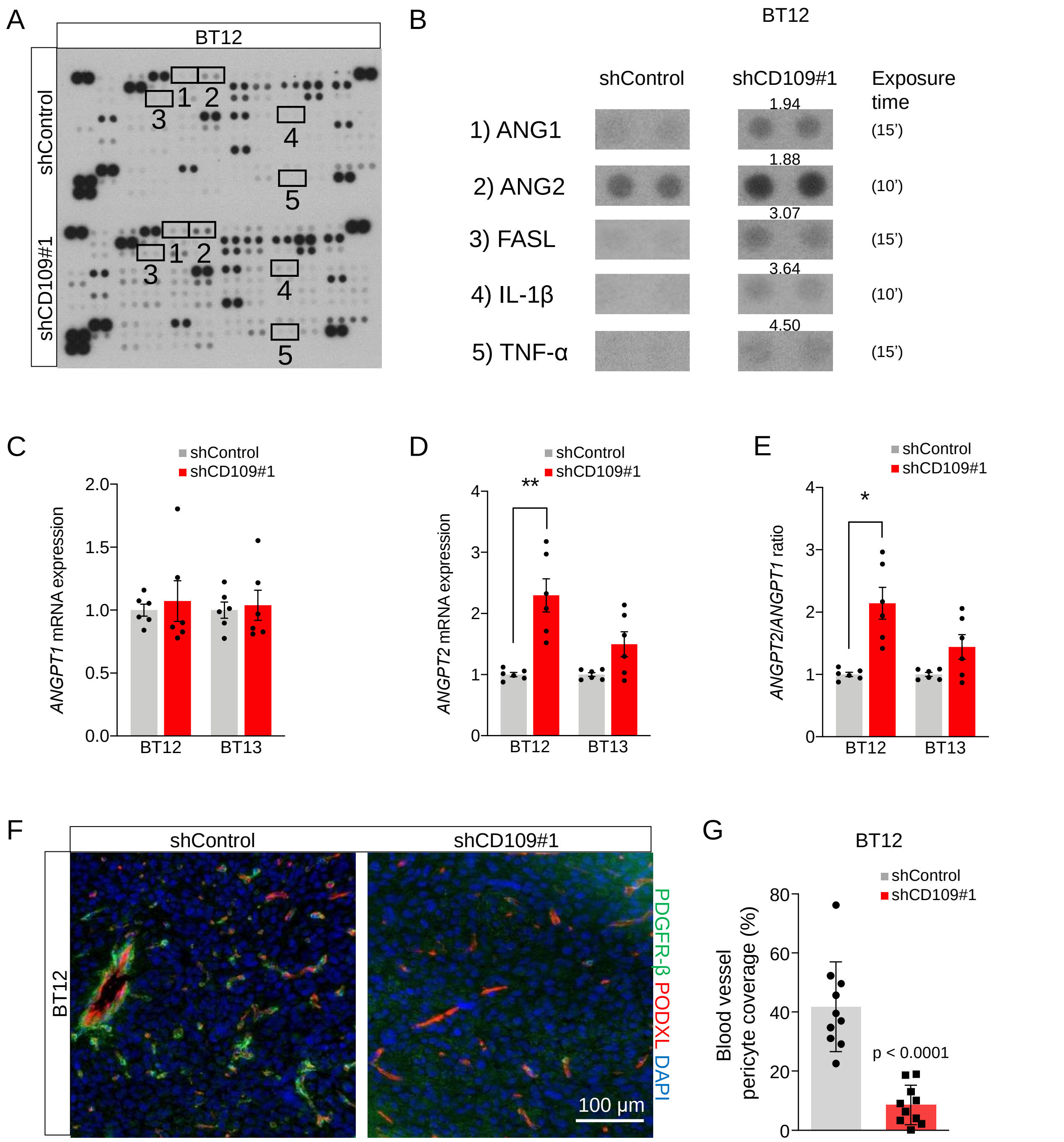

Supplemental Figure 6. CD109 silencing alters the dialogue between glioblastoma cells and the tumor stroma. (**A**) Dot blot of the cell culture medium of non-targeted control (top) and CD109-silenced (bottom) BT12 GSCs. Cell culture medium was collected five days post-transduction and analyzed on a cytokine array. Differentially expressed proteins are identified by numbered boxed

areas, which are magnified in (**B**). (**B**) Levels of secreted pro-inflammatory cytokines (FASL, IL-1β, TNF-α) and angiogenic factors (ANG-1 and ANG-2) in the culture medium of non-targeted control (left) and CD109-silenced (right) gliospheres. Exposure times are indicated. (**C** and **D)**, qRT-PCR analysis of *ANGPT1* (**C**) and *ANGPT2* (**D**) mRNA levels in CD109-silenced and non-targeted control GSCs. Data are presented as mean ± SEM. $^{**}P < 0.01$, non-parametric Mann-Whitney *U* test. (**E**) *ANGPT2*/*ANGPT1* mRNA ratio in CD109-silenced and non-targeted control GSCs. Data are presented as mean ± SEM. $^{*}P < 0.05$, non-parametric Mann-Whitney *U* test. Data are from $n = 3$ (**C**-**E**) independent experiments. (**F**) Micrographs showing the PDGFR-β+ pericyte coverage (green) on the tumor blood vessels (PODXL, red) in BT12 control (left) and CD109-silenced xenografts. Scale bar: 100 μm. (**G**) Quantification of the PDGFR-β+ tumor pericyte coverage in control and CD109-silenced BT12 xenografts. Data are presented as mean ± SD. $^{****}P < 0.0001$, non-parametric Mann-Whitney *U* test.

Supplemental Table 1. Association of CD109 protein expression in tumor cells of diffusively infiltrating gliomas (grades II-IV). CD109 expression significantly associates with increasing tumor grade. Data related to the Figure 1.

CD109 intensity	Grade II	Grade III	Grade IV	Total	Pearson chi-square
	n (%)	*n (%)*	*n (%)*	*n (%)*	*P-value*
**Tumor Cells**					
0	**6** (12.0)	**1** (4.2)	**19** (7.0)	**26** (7.5)	
1	**31** (62.0)	**13** (54.2)	**108** (39.7)	**152** (43.9)	
2	**12** (24.0)	**10** (41.2)	**111** (40.8)	**133** (38.4)	*P* = 0.007
3	**1** (2.0)	**0** (0.0)	**34** (12.5)	**35** (10.1)	
Total	**50** (14.5)	**24** (6.9)	**272** (78.6)	**346** (100.0)	

Supplemental Table 2. Associations of CD109 protein expression in tumor cells of diffusively infiltrating gliomas (grades II-IV). Data related to the Figure 1.

**CD109 intensity**	**0**	**1**	**2**	**3**	**Total**	**Pearson chi-square**
	n (%)	*n (%)*	*n (%)*	*n (%)*	*n (%)*	*P-value*
**Age**						
<60	**15** (65.2)	**61** (52.1)	**39** (40.6)	**9** (31.0)	**124** (46.8)	
>60	**8** (34.8)	**56** (47.9)	**57** (59.3)	**20** (69.0)	**141** (53.2)	
Total	**23** (8.7)	**117** (44.2)	**96** (36.2)	**29** (10.9)	**265** (100.0)	*P* = 0.032
**p53**						
<5%	**5** (23.1)	**23** (21.1)	**13** (14.6)	**4** (16.0)	**45** (18.3)	
≥5%	**18** (78.3)	**86** (78.9)	**76** (85.4)	**21** (84.0)	**201** (81.7)	
Total	**23** (9.3)	**100 (**44.3)	**89** (36.2)	**25** (10.2)	**246** (100.0)	*P* = 0.647
***EGFR* amplification**						
No	**8** (50.0)	**47** (61.8)	**49** (62.0)	**14** (63.6)	**118** (61.1)	
Yes	**8** (50.0)	**29** (38.2)	**30** (38.0)	**8** (36.4)	**75** (38.9)	
Total	**16** (8.3)	**76** (39.4)	**79** (40.9)	**22** (11.4)	**193** (100.0)	*P* = 0.817
**EGFR intensity**						
0	**1** (14.3)	**6** (12.0)	**7** (22.6)	**0** (0.0)	**14** (14.6)	
1	**2** (28.6)	**15** (30.0)	**4** (12.9)	**5** (62.5)	**26** (27.1)	
2	**3** (42.9)	**23** (46.0)	**15** (48.4)	**2** (25.0)	**43** (44.8)	
3	**1** (14.3)	**6** (12.0)	**5** (16.1)	**1** (12.5)	**13** (13.5)	
Total	**7** (7.3)	**50** (52.1)	**31** (32.3)	**8** (8.3)	**96** (100.0)	*P* = 0.352
***IDH1* mutation**						
No	**14** (77.8)	**78** (85.7)	**70** (88.6)	**16** (94.1)	**178** (86.8)	
Yes	**4** (22.2)	**13** (14.3)	**9** (11.4)	**1** (5.9)	**27** (13.2)	
Total	**18** (8.8)	**91** (44.4)	**79** (38.5)	**17** (8.3)	**205** (100.0)	*P* = 0.494
**Ki-67**						
0-4%	**5** (71.4)	**13** (25.0)	**7** (20.0)	**1** (11.1)	**26** (25.2)	
5-10%	**0** (0.0)	**19** (36.5)	**13** (37.1)	**1** (11.1)	**33** (32.0)	
>11%	**2** (28.6)	**20** (38.5)	**15** (42.9)	**7** (77.8)	**44** (42.7)	
Total	**7** (6.8)	**52** (50.5)	**35** (34.0)	**9** (8.7)	**103** (100.0)	*P* = 0.027
**pSTAT3 intensity**						
0	**6** (85.7)	**10** (21.3)	**9** (27.3)	**0** (0.0)	**25** (25.8)	
1	**0** (0.0)	**12** (25.5)	**11** (33.3)	**5** (50.0)	**28** (28.9)	
2	**1** (14.3)	**25** (53.2)	**13** (39.4)	**5** (50.0)	**44** (45.4)	
Total	**7** (7.2)	**47** (48.5)	**33** (34.0)	**10** (10.3)	**97** (100.0)	*P* = 0.004
**Tumor recurrence**						
Primary	**8** (80.0)	**59** (86.8)	**44** (80.0)	**11** (100.0)	**122** (84.7)	
Recurrent	**2** (20.0)	**9** (13.2)	**11** (20.0)	**0** (0.0)	**22** (15.2)	
Total	**10** (69.4)	**68** (47.2)	**55** (38.2)	**11** (76.3)	**144** (100.0)	*P* = 0.345

Supplemental Table 3. Associations of CD109 protein expression in tumor cells of glioblastomas alone (grade IV). Data related to the Figure 1.

**CD109 intensity**	**0**	**1**	**2**	**3**	**Total**	**Pearson chi-square**
	n (%)	*n (%)*	*n (%)*	*n (%)*	*n (%)*	*P -value*
**Age**						
<60	**9** (52.9)	**34** (40.0)	**30** (35.3)	**9** (31.0)	**82** (38.0)	
>60	**8** (47.1)	**51** (60.0)	**55** (64.7)	**20** (69.0)	**134** (62.0)	
Total	**17** (7.9)	**85** (39.4)	**85** (39.4)	**29** (13.4)	**216** (100.0)	*P* = 0.454
**p53**						
<5%	**3** (17.6)	**17** (21.0)	**12** (15.2)	**4** (16.0)	**36** (17.8)	
≥5%	**14** (82.4)	**64** (79.0)	**67** (84.8)	**21** (84.0)	**166** (82.2)	
Total	**17** (8.4)	**81** (40.1)	**79** (39.1)	**25** (12.4)	**202** (100.0)	*P* = 0.805
***EGFR* amplification**						
No	**8** (50.0)	**42** (60.0)	**46** (60.5)	**14** (63.6)	**110** (59.8)	
Yes	**8** (50.0)	**28** (40.0)	**30** (39.5)	**8** (36.4)	**74 (**40.2)	
Total	**16** (8.7)	**70** (38.0)	**76** (41.3)	**22** (12.0)	**184** (100.0)	*P* = 0.851
**EGFR intensity**						
0	**1** (20.0)	**5** (14.3)	**7** (24.1)	**0** (0.0)	**13** (16.9)	
1	**1** (20.0)	**8** (22.9)	**3** (10.3)	**5** (62.5)	**17** (22.1)	
2	**3** (60.0)	**17** (48.6)	**14** (48.3)	**2** (25.0)	**36** (46.8)	
3	**0** (0.0)	**5** (14.3)	**5** (17.2)	**1** (12.5)	**11** (14.3)	
Total	**5** (6.5)	**35** (45.5)	**29** (37.7)	**8** (10.4)	**77** (100.0)	*P* = 0.207
***IDH1* mutation**						
No	**15** (93.8)	**79** (100.0)	**75** (96.2)	**27** (96.4)	**196** (97.5)	
Yes	**1** (6.3)	**0** (0.0)	**3** (3.8)	**1** (3.6)	**5** (2.5)	
Total	**16** (8.0)	**79** (39.3)	**78** (38.8)	**28 (**13.9)	**201** (100.0)	*P* = 0.298
**Ki-67**						
0-4%	**6** (46.2)	**11** (15.1)	**12** (16.7)	**2** (13.3)	**31** (17.9)	
5-10%	**1** (7.7)	**23** (31.5)	**27** (37.5)	**2** (13.3)	**53** (30.6)	
>11%	**6** (46.2)	**39** (53.4)	**33** (45.8)	**11** (73.3)	**89** (51.4)	
Total	**13** (7.5)	**73** (42.2)	**72** (41.6)	**15** (8.7)	**173** (100.0)	*P* = 0.041
**pSTAT3 intensity**						
0	**5** (100.0)	**6** (18.8)	**7** (22.6)	**0** (0.0)	**18** (23.1)	
1	**0** (0.0)	**10** (31.3)	**11** (35.5)	**5** (50.0)	**26** (33.3)	
2	**0** (0.0)	**16** (50.0)	**13** (41.9)	**5** (50.0)	**34** (43.6)	
Total	**5** (6.4)	**32 (**41.0)	**31** (39.7)	**10** (12.8)	**78** (100.0)	*P* = 0.002
